# High-performance silicon−graphene hybrid plasmonic waveguide photodetectors beyond 1.55 μm

**DOI:** 10.1038/s41377-020-0263-6

**Published:** 2020-02-28

**Authors:** Jingshu Guo, Jiang Li, Chaoyue Liu, Yanlong Yin, Wenhui Wang, Zhenhua Ni, Zhilei Fu, Hui Yu, Yang Xu, Yaocheng Shi, Yungui Ma, Shiming Gao, Limin Tong, Daoxin Dai

**Affiliations:** 1 State Key Laboratory for Modern Optical Instrumentation, Zhejiang Provincial Key Laboratory for Sensing Technologies, College of Optical Science and Engineering, International Research Center for Advanced Photonics, Zhejiang University, Zijingang Campus, 310058 Hangzhou, China; 2 Ningbo Research Institute, Zhejiang University, 315100 Ningbo, China; 3 Department of Physics and Key Laboratory of MEMS of the Ministry of Education, Southeast University, 211189 Nanjing, China; 4 College of Information Science and Electronic Engineering, Zhejiang University, 310027 Hangzhou, Zhejiang China

**Keywords:** Optical properties and devices, Silicon photonics, Integrated optics

## Abstract

Graphene has attracted much attention for the realization of high-speed photodetection for silicon photonics over a wide wavelength range. However, the reported fast graphene photodetectors mainly operate in the 1.55 μm wavelength band. In this work, we propose and realize high-performance waveguide photodetectors based on bolometric/photoconductive effects by introducing an ultrathin wide silicon−graphene hybrid plasmonic waveguide, which enables efficient light absorption in graphene at 1.55 μm and beyond. When operating at 2 μm, the present photodetector has a responsivity of ~70 mA/W and a setup-limited 3 dB bandwidth of >20 GHz. When operating at 1.55 μm, the present photodetector also works very well with a broad 3 dB bandwidth of >40 GHz (setup-limited) and a high responsivity of ~0.4 A/W even with a low bias voltage of −0.3 V. This work paves the way for achieving high-responsivity and high-speed silicon–graphene waveguide photodetection in the near/mid-infrared ranges, which has applications in optical communications, nonlinear photonics, and on-chip sensing.

## 2 µm photodetectors: silicon−graphene−metal hybrid plasmonics

The use of a silicon−graphene plasmonic waveguide has enabled the realization of fast and sensitive photodetectors that operate at the wavelength of 2 µm. In order to satisfy the demands for the applications in optical communication and optical sensing, there is the need to extend silicon photonics to wavelengths beyond 1.55 µm. However, it is a challenge to create high-performance photodetectors at these wavelengths. Now, Daoxin Dai and coworkers from Zhejiang University and Southeast University in China have proposed and realized a silicon−graphene hybrid plasmonic waveguide photodetector that operates at 2 µm with a responsivity of ~70 mA/W and a 3-dB bandwidth over 20 GHz. In this design, efficient light absorption in graphene is enabled by using a hybrid plasmonic waveguide with a wide thin silicon ridge core and a metal cap that serves as a signal electrode.

## Introduction

Currently, it is desirable to extend the wavelength band of silicon photonics^1^ beyond 1.55 μm, e.g., 2 μm, for many important applications in optical communications^2,3^, nonlinear photonics^4^, and on-chip sensing^5–7^. However, the realization of high-performance silicon-based waveguide photodetectors beyond 1.55 μm still faces challenges. For example, the reported GeSn^8^ and ion-implanted silicon^9^ photodetectors still operate in the limited wavelength band of <2.5 μm, while III−V photodetectors^10^ are unsuitable for monolithic integration on silicon. As an alternative, two-dimensional materials^11,12^ (e.g., graphene^13,14^ and black phosphorus^15^) provide a promising solution because of their broad operation wavelength band and advantage of avoiding material and structure mismatch in the design and fabrication. At present, black-phosphorus photodetectors have limited bandwidths of ~3 GHz^16–18^, and their fabrication is not easy. In contrast, large-size graphene sheets are commercially available and can be transferred/patterned easily in the wafer process line^19^. Recently, several fast silicon−graphene waveguide photodetectors at 1.31/1.55 μm have been reported with a high bandwidth of ~100 GHz^20,21^. Among these photodetectors, the metal−graphene−metal (MGM) configuration is widely used, since the high mobility of graphene facilitates high-speed operation. However, MGM graphene photodetectors^19–32^ usually have limited responsivities when operating at low bias voltages. For example, in ref. ^20^, the reported responsivities are <170 mA/W at −0.4 V and <400 mA/W at −0.6 V for *mono*-layer and *bi*-layer graphene photodetectors, respectively. In addition, for the 40 GHz graphene-semiconductor heterostructure (GSH) photodetector reported recently^33^, the responsivity is also very low (~11 mA/W). More recently, a graphene-insulator-graphene (GIG) photodetector was reported with an improved responsivity of 0.24 A/W and an estimated 3 dB bandwidth of 56 GHz. Unfortunately, the working bias voltage is as high as 10 V^34^. Therefore, high-speed and high-responsivity graphene photodetectors with low bias voltages are still highly desired. Notably, very few results have been reported for the realization of graphene waveguide photodetectors beyond 1.55 μm, even though light absorption in graphene is present in this range. For the reported surface-illuminated mid-IR graphene photodetectors^35–40^, the responsivity is low due to the limited light absorption, which is well known. For the mid-IR graphene waveguide photodetectors reported in recent years^41–43^, the measured bandwidths are very limited (e.g., several hundreds of kHz or less). To the best of our knowledge, currently, high-speed (e.g., >10 GHz) silicon−graphene waveguide photodetectors have not been reported for the mid-IR range beyond the wavelength band of 1.55 μm.

In this paper, we propose and demonstrate high-speed and high-responsivity silicon−graphene waveguide photodetectors beyond 1.55 μm by utilizing a hybrid plasmonic waveguide with an ultrathin wide silicon ridge. With this novel design, the light absorption in graphene is enhanced while the metal absorption loss is reduced simultaneously, which helps to greatly improve the responsivity. Here, the wide metal cap in the middle and the MGM sandwiched structures are introduced as the signal electrode and the ground electrodes, respectively, so that one can achieve reduced graphene-metal contact resistances (e.g., several tens of ohms) and a large 3 dB bandwidth. A mechanism analysis confirms that the photothermoelectric (PTE) effect dominates the photoresponse under zero bias, while the bolometric (BOL)/photoconductive (PC) effects become dominant when a bias voltage is applied. When operating at 2 μm, the present graphene photodetector has a responsivity of ~70 mA/W and a measured 3 dB bandwidth of >20 GHz (which is setup-limited). Meanwhile, the present photodetectors also work very well at 1.55 μm. The measured responsivity is approximately 0.4 A/W for a bias voltage of −0.3 V and an optical power of 0.16 mW, while the 3 dB bandwidth is over 40 GHz (setup-limited).

## Results

### Structure and design

**Fig. 1:**
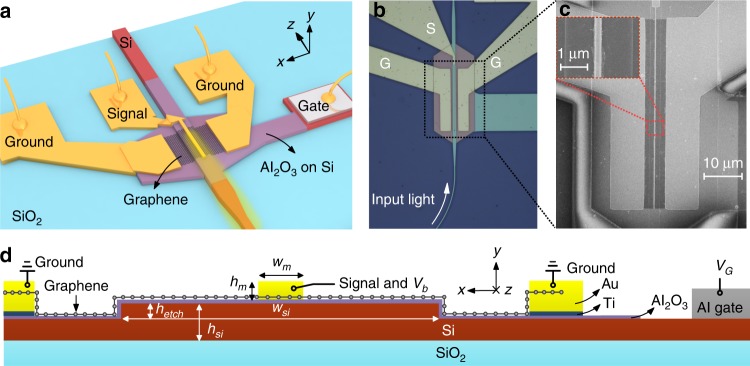
Structures of the present silicon−graphene hybrid plasmonic waveguide photodetector. **a** Schematic configuration. **b** Optical microscopy image. **c** SEM images. **d** Cross-section of the present silicon−graphene hybrid plasmonic waveguide with the signal electrode in the middle and the ground electrodes on both sides (here, the metal−graphene−metal sandwich structure is utilized). *V*_b_ bias voltage, *V*_G_ gate voltage

Figure 1a, b shows the configuration of the present silicon−graphene hybrid plasmonic waveguide photodetector, which consists of a passive input section based on a silicon-on-insulator (SOI) strip waveguide and an active region based on a silicon−graphene hybrid plasmonic waveguide. These two parts are connected through a mode converter based on a lateral taper structure. As shown in Fig. 1c, the present hybrid plasmonic waveguide has a silicon ridge core region, an ultrathin Al_2_O_3_ insulator layer, a graphene sheet, and a metal cap. The metal cap in the middle is used as the signal electrode, while the ground electrodes are placed far away from the silicon ridge to avoid high metal absorption loss. In particular, here, we introduce the MGM sandwiched structure for the ground electrodes in order to achieve reduced graphene-metal contact resistances, which helps achieve a large 3 dB bandwidth^44^. For previous silicon−graphene hybrid plasmonic waveguide photodetectors, the center metal strip exhibits high absorption of light even though the light−graphene interaction can be enhanced^24,27^, in which case the undesired metal absorption without any contribution to the photocurrent generation is even higher than the desired graphene absorption. As a result, the responsivity is usually limited^24,27^. This problem can be alleviated partially by reducing the width of the center metal strip (e.g., 70 nm^27^). However, this reduction in width introduces a high graphene-contact resistance, which consequently leads to a reduction in the responsivity and the bandwidth. In this paper, a silicon−graphene hybrid plasmonic waveguide is proposed with a wide silicon ridge, as shown in Fig. 1d. For the present waveguide, the silicon core layer is chosen to be as thin as 100 nm instead of the regular thickness of 220 nm^19,28,29^ so that the light absorption in graphene is enhanced due to the weak mode field confinement in the vertical direction^45^. Furthermore, for the hybrid photonic-plasmonic mode^46^ supported in the present waveguide, the metal absorption loss is low even when a relatively wide metal strip is chosen to achieve a low metal−graphene-contact resistance. Meanwhile, the center metal strip (the signal electrode) on top of the silicon ridge still helps improve the light absorption in graphene due to the strong localized field. In this way, the present hybrid plasmonic waveguide can simultaneously realize low metal loss and high absorption in graphene. In addition, the silicon ridge height is chosen to be as small as 50 nm, which helps to avoid damage to the graphene sheet during the fabrication processes. As shown in Fig. 1, an Al gate electrode is integrated on top of the silicon slab region; thus, the silicon ridge acts as a global gate electrode. In this way, one can manipulate the graphene chemical potential by applying a gate bias voltage, as proposed in ref. ^24^ and demonstrated in refs. ^29,30^.

**Fig. 2:**
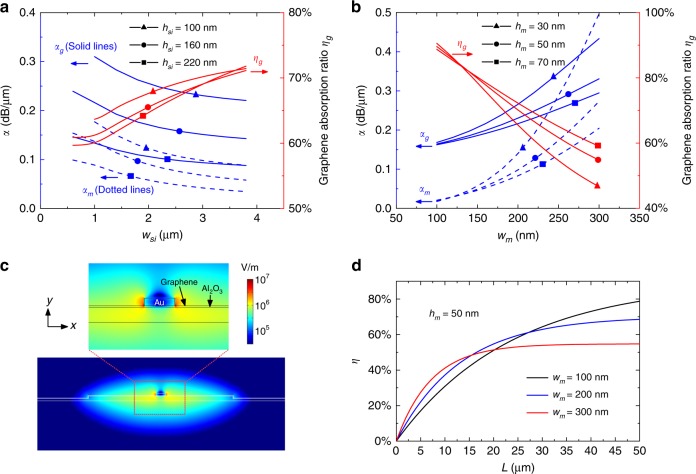
Mode properties of the present silicon−graphene hybrid plasmonic waveguide when operating at λ = 2 μm. **a** Calculated absorption coefficients (*α*_g_, *α*_m_) and the graphene absorption ratio *η*_g_ as the silicon ridge width *w*_si_ varies for cases with different silicon ridge heights *h*_si_. Here, *w*_m_ = 200 nm, and *h*_m_ = 50 nm. **b** Calculated absorption coefficients (*α*_g_, *α*_m_) and the graphene absorption ratio *η*_g_ as the metal strip width *w*_m_ varies for cases with different metal heights *h*_m_. Here, *w*_si_ = 3 μm, and *h*_si_ = 100 nm. **c** The electric field component $\sqrt {\left| {\overrightarrow {E_x} } \right|^2 + \left| {\overrightarrow {E_z} } \right|^2}$ distribution of the quasi-TE_0_ mode for the optimized silicon−graphene hybrid plasmonic waveguide. **d** Calculated graphene absorptance *η* as the propagation length *L* varies for cases with different metal widths of *w*_m_ = 100, 200, and 300 nm. Here, *h*_m_ = 50 nm, *w*_si_ = 3 μm, and *h*_si_ = 100 nm

Note that the thin-silicon photonic waveguide and the silicon−graphene hybrid plasmonic waveguide are polarization-sensitive. Here, we consider the case of TE polarization; thus, a TE-type grating coupler is used to achieve efficient fiber-to-chip coupling. The input light is coupled to the TE_0_ mode of the thin-silicon photonic waveguide and then coupled to the quasi-TE_0_ mode of the silicon−graphene hybrid plasmonic waveguide^46^ with a low coupling loss. Figure 2a, b shows the calculation results of evaluating the light absorption induced by the graphene sheet and the metal strip for the quasi-TE_0_ mode in the present silicon−graphene hybrid plasmonic waveguide as the waveguide dimensions vary. Here, a finite-element method mode-solver tool (COMSOL) is used (see more details in Supplementary Note 1). The graphene absorptance is given by *η*(*L*) = *η*_g_(1 − 10^−0.1*αL*^), where *L* is the propagation distance, *α* is the mode absorption coefficient in dB/μm, *η*_g_ is the ratio of the graphene absorption to the total absorption, i.e., $\eta _{\mathrm{g}} = \frac{{\alpha _{\mathrm{g}}}}{\alpha } = \frac{{\alpha _{\mathrm{g}}}}{{\alpha _{\mathrm{g}} + \alpha _m}}$ (here, *α*_g_ and *α*_m_ are the absorption coefficients of the graphene sheet and the metal strip, respectively). Since only the graphene absorption contributes to the photocurrent, one should maximize the ratio *η*_g_ so that the graphene absorption is more dominant than the metal absorption to improve the responsivity. Figure 2a shows the absorption ratio *η*_g_ and the results for the absorption coefficients (*α*_g_, *α*_m_) as the ridge width *w*_si_ varies from 0.5 to 4.0 μm. Here, the width and height of the metal strip are chosen as *w*_m_ = 200 nm and *h*_m_ = 50 nm, respectively. As shown in Fig. 2a, the graphene absorption ratio *η*_g_ increases when choosing a wider ridge. When the ridge width *w*_si_ is chosen to be larger than 3 μm, the ratio *η*_g_ is higher than 70%. Meanwhile, it is noted that the absorption coefficients (*α*_g_, *α*_m_) decrease when choosing a wider ridge, which is simply due to more optical confinement in the silicon region and weaker light−matter interaction in the absorption regions. As a result, one needs to choose a longer absorption length to achieve sufficient absorption in the photodetector, which prevents fast responses due to the RC-constant limitation. Fortunately, the light absorption can be enhanced greatly by reducing the silicon core height *h*_si_, as shown in Fig. 2a, where the absorption coefficients (*α*_g_, *α*_m_) for the cases with different silicon core heights of *h*_si_ = 220, 160, and 100 nm are given. From this figure, one sees that the absorption coefficients *α*_g_ and *α*_m_ increase by more than 100% when the core height *h*_si_ is reduced from 220 to 100 nm. This result is attributed to the stronger evanescent field for the case with a thinner silicon core. Meanwhile, the graphene absorption ratio *η*_g_ increases slightly as the core height *h*_si_ decreases. As a result, an ultrathin silicon core is preferred to achieve strong light absorption so that one can use a short absorption section. Here, we choose *h*_si_ = 100 nm for our devices based on the feasibility of the fabrication processes. To avoid a long carrier transit time between the electrodes, the ridge width is chosen as *w*_si_ = 3 μm. With this design, the absorption coefficients are (*α*_g_, *α*_m_) = (0.230, 0.098) dB/μm, and the graphene absorption ratio *η*_g_ is approximately 70%.

Figure 2b shows the dependence of the ratio *η*_g_ and the absorption coefficients (*α*_g_, *α*_m_) on the width *w*_m_ and height *h*_m_ of the metal strip. Here, the dimensions of the silicon ridge are *w*_si_ = 3 μm and *h*_si_ = 100 nm. It can be seen that a high ratio *η*_g_ can be achieved by choosing a narrow metal strip, which is simply due to a significant reduction in the metal absorption. For example, when choosing *w*_m_ = 100 nm, the metal absorption coefficient is as small as *α*_m_ = 0.019 dB/μm, while the ratio *η*_g_ is as high as ~90%. However, the graphene absorption coefficient *α*_g_ also decreases to some degree when the metal strip becomes narrow. Therefore, to have a sufficiently high graphene absorption coefficient and a high absorption ratio *η*_g_, we choose *w*_m_ = 200 nm in our design, which also makes the fabrication relatively easy and guarantees a low graphene-metal contact resistance for the middle electrode. The absorption coefficients (*α*_g_, *α*_m_) can also be further enhanced by reducing the metal thickness, as shown in Fig. 2b. However, the graphene absorption ratio *η*_g_ also decreases. Therefore, we choose *h*_m_ = 50 nm as a trade-off.

For the designed silicon−graphene hybrid plasmonic waveguide with *w*_m_ = 200 nm, *h*_m_ = 50 nm, *w*_si_ = 3 μm, and *h*_si_ = 100 nm, the calculated electric field distribution $\sqrt {\left| {\overrightarrow {E_x} } \right|^2 + \left| {\overrightarrow {E_z} } \right|^2}$of the quasi-TE_0_ mode is shown in Fig. 2c. It can be seen that there is strong field localization and enhancement in the area around the metal strip. For example, the electric field component $\sqrt {\left| {\overrightarrow {E_x} } \right|^2 + \left| {\overrightarrow {E_z} } \right|^2}$ along the graphene layer at the metal corners reaches up to 1.0 × 10^7^ V/m for 1 mW optical power, which helps enhance the light absorption in graphene. For the present design, we calculate the total graphene absorption *η*(*L*) as the propagation distance *L* varies from 0 to 50 μm, as shown in Fig. 2d. It can be seen that the total graphene absorptance is almost saturated at approximately 68.6% for the case of *w*_m_ = 200 nm when the length *L* is 50 μm. For a metal width of *w*_m_ = 300 nm, the total graphene absorptance is close to a saturated value of 51.4% when the length *L* is 20 μm, which occurs because the metal absorption increases. In contrast, when *w*_m_ = 100 nm, the total graphene absorption increases to 78.7% (not yet saturated) when the length *L* increases to 50 μm, which is due to the relatively low absorption coefficients (*α*_g_, *α*_m_). With such a design, the present silicon−graphene hybrid plasmonic waveguide achieves the best result among the results of the reported silicon−graphene hybrid waveguides (which were developed for 1.55 μm). For a direct comparison, the silicon−graphene hybrid plasmonic waveguide is also designed optimally for 1.55 μm (see Supplementary Note 1), and the graphene absorptance at 1.55 μm is approximately 54.3% for the optimal design with *w*_m_ = 200 nm when the length *L* = 20 μm. In contrast, in ref. ^24^, the graphene absorptance is 44% only for the *bi*-layer-graphene hybrid plasmonic waveguide with *w*_m_ = 180 nm and *L* = 22 μm. For the Si_3_N_4_-graphene hybrid plasmonic waveguide with *w*_m_ = 70 nm in ref. ^27^, the graphene absorptance *η* is 42% when the length is *L* = 40 μm. More recently, a plasmonic-enhanced graphene waveguide with bowtie-shaped metallic structures was reported with a short device length of 6 μm; however, the graphene absorptance is saturated at ~34%^20^.

### Measurement results and analyses

The designed waveguide photodetectors were fabricated with a series of steps (see Methods), including the processes of electron-beam lithography, ICP etching, Al_2_O_3_ atom-layer deposition, graphene transfer, and metal deposition. For the fabricated devices, the *I*−*V* characteristics were characterized by varying the gate voltage (see Supplementary Note 2). The contact resistance and the graphene properties were obtained by fitting the measured resistance data with a simple capacitance model^29^. The typical value for the graphene mobility is ~500 cm^2^/V s, which is not as high as the best results reported by some other groups^28,29^. This might be due to the defects introduced during the fabrication processes; the graphene mobility could possibly be enhanced further by improving the fabrication processes in the future. For all of the devices, the total contact resistances are typically several tens of Ohms, depending on the sizes of the contact regions and some random variations introduced in the fabrication processes. As an example, the total contact resistance is approximately 45 Ω for Device A, which is characterized in more detail in the following sections.

**Fig. 3:**
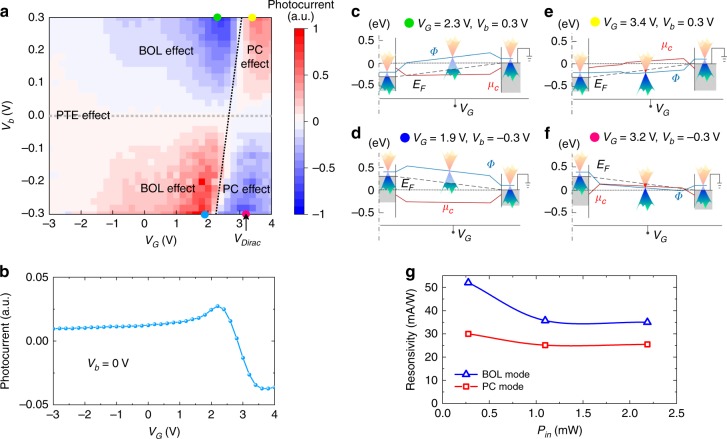
Static characterization of the present silicon−graphene hybrid plasmonic waveguide photodetector (Device A). **a** Measured photocurrent map as the gate voltage *V*_G_ and the bias voltage *V*_b_ vary. **b** Dependence of the photocurrent at zero bias for the gate voltage *V*_G_. **c**−**f** Calculated energy band diagrams for the cases of (*V*_G_, *V*_b_) = (2.3, 0.3), (1.9, −0.3), (3.4, 0.3), and (3.2, −0.3) V. **g** Measured responsivities with different input optical powers *P*_in_. Here, *V*_b_ = −0.3 V and *V*_G_ = ~1.9 V (for the BOL effect), or *V*_G_ = ~3.2 V (for the PC effect)

The photocurrents were measured by using a lock-in amplifier (see Methods and Supplementary Note 7). The gate voltage *V*_G_ is set to less than 4.0 V to avoid the breakdown of the Al_2_O_3_ nanolayer. Figure 3a shows the measured photocurrent map for one of the representative devices (Device A) operating with different gate voltages *V*_G_ and bias voltages *V*_b_. For Device A, the Dirac voltage *V*_Dirac_ is approximately 3.2 V (see the measurement in Supplementary Note 2). The photocurrent map has a fourfold pattern, which is similar to the measured results for the device reported in ref. ^28^, even though the structural designs of the devices are different. From this figure, it can be seen that the photocurrent strongly depends on the gate voltage *V*_G_ and the bias voltage *V*_b_. To see more details, the dependence of the photocurrent at zero bias for the gate voltage *V*_G_ is shown in Fig. 3b, which shows that there is a transition from a positive photocurrent to a negative photocurrent when the gate voltage *V*_G_ is approximately 2.7 V. It is well known that such behavior for the dependence of the photocurrent on the gate voltage *V*_G_ is very typical for the PTE photocurrent^47,48^. Our photocurrent modeling in Supplementary Note 6 (see Supplementary Fig. S7d) further confirms that the PTE effect is the dominant mechanism for the zero-bias photocurrent. As shown by the fourfold pattern in Fig. 3a, when the bias voltage *V*_b_ is applied, the photocurrent increases greatly, which indicates that the PTE effect is no longer the dominant mechanism. The reason is that the PTE photocurrent is generally not sensitive to the bias voltage *V*_b_, as observed previously^29^. This result is also predicted by the theoretical modeling in Supplementary Note 6. Instead, the dominant mechanisms for generating the photocurrent are very likely to be the BOL effect or the PC effect when *V*_b_ ≠ 0. As shown in Fig. 3a, the fourfold photocurrent map has two subparts, i.e., the left and right regions divided by the dotted line located around *V*_G_ = 2.3−3 V. On the left side, the signs for the measured photocurrent and the bias voltage are opposite, which indicates that the dominant mechanism is the BOL effect^49^. In contrast, on the right side, the signs for the photocurrent and the bias voltage are consistent, which indicates that the dominant mechanism is the PC effect^29^.

In order to better understand the mechanisms of the photodetectors, we also provide theoretical calculations for the Fermi level *E*_F_, the Dirac-point energy *Φ*, and the chemical potential *μ*_c_ along the graphene channel between the signal electrode and the right ground electrode (see the details in Supplementary Note 5), as shown in Fig. 3c–f. In this calculation, the bias voltage is chosen to be *V*_b_ = ±0.3 V, while the gate voltage is chosen as *V*_G_ = ~2.0 and ~3.2 V, located on the left and right sides of the photocurrent map (see the labels in Fig. 3a). Here, the chemical potential for the graphene sheet underneath the gold electrodes is estimated to be approximately −0.1 eV due to the pinning effect^50^. In contrast, the chemical potential of the graphene sheet in the channel center is fully gate-controllable, and there is a transition region gradually varying from the pinning region and the fully gate-controllable region. As shown in Fig. 3c, d, which correspond to the cases with (*V*_G_, *V*_b_) = (2.3, 0.3) V and (1.9, −0.3) V, respectively, the graphene sheet is highly doped. As a result, the bolometric coefficient *β* is large^11,49^; thus, the BOL effect becomes the dominant mechanism. In Fig. 3e, f, which correspond to the cases with (*V*_G_, *V*_b_) = (3.4, 0.3) V and (3.2, −0.3) V, respectively, the graphene sheet is lightly doped. As a result, the bolometric coefficient *β* is small^11,49^; thus, the BOL effect is suppressed. Meanwhile, the lifetime of the photogenerated carriers in graphene becomes long because of the low doping level^49^. In this case, the density of the photogenerated carriers is sufficiently high, and the PC effect becomes the dominant mechanism for the photoresponse.

In summary, when the bias voltage |*V*_b_| increases from 0 to 0.3 V, the dominant mechanism for the photoresponse changes from the PTE effect to the BOL effect or the PC effect, depending on the applied gate voltage. Meanwhile, the responsivity increases significantly if the gate voltage is controlled well. Figure 3g shows the measured responsivity for Device A operating with *V*_b_ = −0.3 V when choosing *V*_G_ = ~1.9 V (the BOL effect) and ~3.2 V (the PC effect). The responsivities for the BOL and PC modes are 35.0 and 25.5 mA/W, respectively, when the input optical power *P*_in_ is ~2.2 mW. When the input optical power *P*_in_ decreases to 0.28 mW, the responsivities increase to approximately 52.1 and 30.0 mA/W for the BOL mode (*V*_G_ = ~1.9 V) and the PC mode (*V*_G_ = ~3.2 V), respectively. Since MGM-type graphene photodetectors often have a high dark current (see the *I*−*V* curves in Supplementary Fig. S9a), the signal-to-dark-current ratio is usually relatively low^19–30,43,49^ in the absence of photoconductive gain. As shown by the noise analysis presented in Supplementary Note 8, the noise equivalent powers (NEPs) of Device A are 6.68−9.92 × 10^2^ pW/Hz^1/2^ and 61.7−72.7 pW/Hz^1/2^ for the BOL and PC modes, respectively, when *P*_in_ = 0.28−2.2 mW. It can be seen that the PC mode achieves a better sensitivity than the BOL mode because of the lower dark current and similar responsivity. In the future, the dark current could be reduced by introducing some junction structures^14^.

**Fig. 4:**
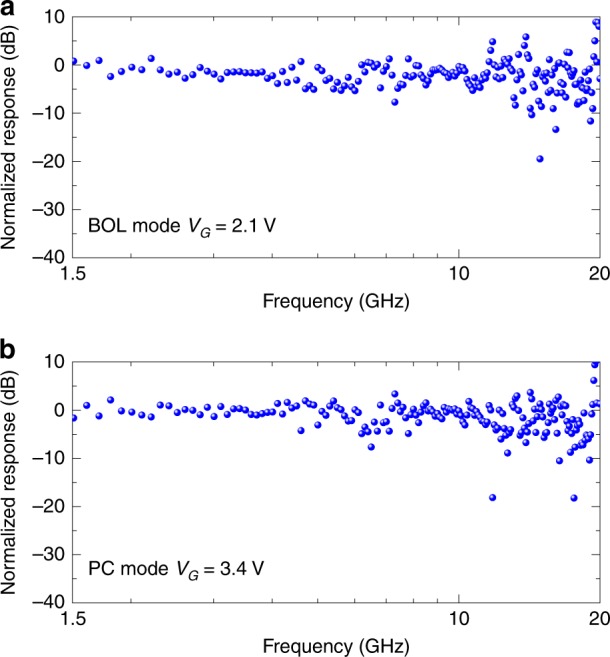
Measured frequency responses of Device A operating at different gate voltages. **a** *V*_G_ = 2.1 V (the BOL mode). **b** *V*_G_ = 3.4 V (the PC mode)

The frequency responses of the devices were measured by using a setup combining a commercial 10 GHz optical modulator and a vector network analyzer (VNA, 40 GHz bandwidth), as shown in Fig. 4a, b. The gate voltages were chosen as *V*_G_ = 2.1 and 3.4 V, corresponding to the BOL effect and the PC effect, respectively. Because the output optical power of the optical modulator at 2 μm is limited and there is no 2 μm optical amplifier available in the lab, the input optical power to the photodetectors is limited to 0.5 mW. In this case, the small-signal photocurrent (on the scale of μA) is much lower than the dark current (~3 mA). Thus, some notable noise was observed at high frequencies in the measurement, as shown in Fig. 4a, b. From this figure, no notable decay is observed in the frequency range of 1.5−20 GHz for both cases with the BOL effect and the PC effect. Here, the maximal frequency *f*_max_ in the measurement is up to 20 GHz, which is limited by the 2 μm optical modulator (with a 3 dB bandwidth of 10 GHz) available in the lab.

**Fig. 5:**
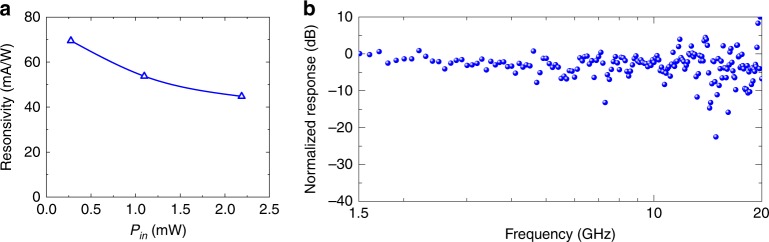
Experimental results for Device B operating at λ = 2 μm. **a** Measured responsivities with different input powers *P*_in_ (*V*_b_ = −0.3 V). **b** Measured frequency response (*V*_b_ = −0.5 V and *V*_G_ = 2.9 V)

Figure 5a, b shows the measured responsivity and the frequency response for another photodetector (Device B) on the same chip. For Device B, the graphene is highly p-doped with a Dirac voltage *V*_Dirac_ larger than 4.0 V (see Supplementary Fig. S3a), which is the maximal gate voltage used in our experiment regarding the breakdown condition of the 10-nm-thick Al_2_O_3_ layer. In this case, Device B operates based on the BOL effect. As shown in Fig. 5a, the responsivity is up to 70 mA/W when *V*_b_ = −0.3 V and *P*_in_ = 0.28 mW. From the measured frequency response shown in Fig. 5b, there is no notable decay in the measured frequency range despite the noise, which shows that the 3 dB bandwidth BW_3 dB_ is also more than 20 GHz.

**Fig. 6:**
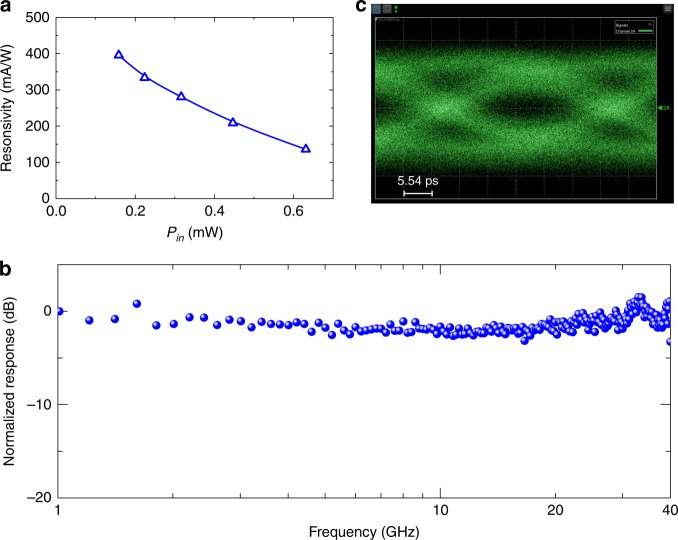
Experimental results for Device C when operating at 1.55 μm. **a** Measured responsivities with different input powers *P*_in_ (*V*_b_ = −0.3 V). **b** Measured frequency response (*V*_b_ = 0.6 V and *V*_G_ = 2.8 V). **c** Measured eye diagram for a 30 Gbps PRBS data stream when *V*_b_ = −1 V and *V*_G_ = 0.3 V

To verify the high bandwidth of the present waveguide photodetector, we characterized the third device (Device C) on the same chip, as shown in Fig. 6a. Device C is very similar to Devices A and B and has a grating coupler for 1.55 μm, so that the high-speed measurement setup for 1.55 μm available in the lab can be used. For Device C with a 20-μm-long absorption length, the Dirac voltage *V*_Dirac_ is higher than 4 V (see Supplementary Fig. S3a), and the BOL effect is the dominant mechanism. From Fig. 6a, Device C has a responsivity of 396 mA/W when *V*_b_ = −0.3 V and *P*_in_ = 0.16 mW. The high responsivity of Device C is attributed to the high light absorption in graphene and thus the high light-induced temperature increase (which is beneficial for achieving a high bolometric photoresponse). Figure 6b shows the measured frequency response of Device C operating at *V*_b_ = 0.6 V, which was characterized with the help of an erbium-doped fiber amplifier at 1.55 μm. The measured 3 dB bandwidth is higher than 40 GHz (which is the maximal bandwidth of our VNA). This device was further used to receive high-bit-rate data with the setup shown in Supplementary Fig. S8d. Figure 6c shows the measured eye diagram for the photodetector operating at 30 Gbit/s when *V*_b_ = 0.6 V and *V*_G_ = 2.8 V. It can be seen that the eye diagram is open with a bit rate as high as 30 Gbit/s. More details are provided in Supplementary Note 7.

### Comparisons

Here, we provide a comprehensive comparison of the performances of the reported silicon−graphene photodetectors beyond 1.55 μm, as shown in Table 1. Several surface-illuminated silicon−graphene photodetectors with broad operation wavelength bands have been reported. In ref. ^35^, a silicon−graphene photodetector was demonstrated with a responsivity of 6.25 mA/W at 10 μm and an estimated 3 dB bandwidth of >1 GHz at 1.03 μm. In ref. ^36^, a silicon−graphene photodetector was reported with responsivities of 0.6−0.076 A/W for an input optical power of 2.5−50 μW. For the device in ref. ^36^, the measured 3 dB bandwidth was higher than 50 GHz at 0.8 μm, and the responsivity was 2−11.5 A/W for an ultralow optical power in the wavelength range of 3−20 μm. For the waveguide photodetector reported recently^35,41–43^, the measured 3 dB bandwidths were on the scale of kHz or not given. In contrast, the present photodetectors (e.g., Device B) have a responsivity of 70 mA/W (at −0.3 V and 0.28 mW) and a setup-limited 3 dB bandwidth of >20 GHz.

**Table 1:** Performances of graphene photodetectors in the mid-infrared range beyond the 1.55 μm wavelength band

Reference	Type	Mechanism	*λ* (μm)	(External) Responsivity	*P*_in_ (μW)	*V*_bias_ (V)	BW_3 dB_
^35^	GSH, surface-illuminated	IPE	2	0.16 mA/W	~0.5	0	~kHz
^36^^a^	MGM, surface-illuminated	PV	3	2 A/W	2.5	0.02	–
^37^^b^	MGM, surface-illuminated (at *T* = 10 K)	BOL	10	6.25 mA/W	0.8	2.4 × 10^−5^	–
^38^	GIG, surface-illuminated	Photogating	Up to 3.2	>1 A/W	~6	1	~Hz
^41^	GSH, waveguide-type	IPE	2.75	130 mA/W	<1	1.5	~11 kHz
^42^	GSH, waveguide-type	IPE	2.75	4.5 mA/W	10	−1	–
^43^	MGM, waveguide-type	–	3.8	2 mA/W	~300	−1	–
This work: Device A	MGM, waveguide-type	BOL	2	52 mA/W	280	−0.3	>20 GHz^c^
This work: Device B				70 mA/W			>20 GHz^c^

^a^The operation wavelength ranges from 0.8 to 20 μm, and the 3 dB bandwidth of 50 GHz is measured at *λ* = 0.8 μm

^b^The operation wavelengths are 0.658, 1.03, 2, and 10 μm. The external responsivity is evaluated from an internal responsivity of 2 × 10^5^ V/W, while the graphene absorptance is 0.5%. The 3 dB bandwidth of >1 GHz is measured at 1.03 μm

^c^The 3 dB bandwidths are setup-limited

**Fig. 7:**
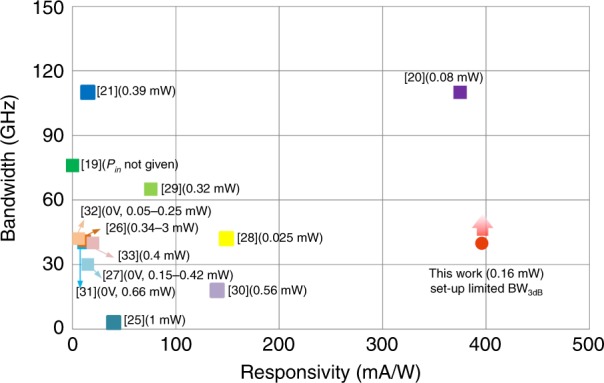
Comprehensive comparisons of previously reported GHz graphene photodetectors at 1.55 μm. The data are for |*V*_b_ | = 0.3 V unless indicated. In refs. ^20,21,28,30^, the responsivities at |*V*_b_ | = 0.3 V are estimated according to the data given in the literature

We further compare the reported silicon−graphene photodetectors in a wavelength band of 1.55 μm, because abundant measurement results are reported in this band, as shown in Fig. 7. Here, only devices with a monolayer graphene sheet and a 3 dB bandwidth of >1 GHz are included. It can be seen that a number of results with high bandwidths of >40 GHz were reported^19–21,26,28,29,31–33^. More recently, the device demonstrated in ref. ^20^ showed a 3 dB bandwidth of over 110 GHz and 100 Gbps data reception. Similarly, the present silicon−graphene hybrid waveguide photodetector also demonstrates a high 3 dB bandwidth of >40 GHz, which is setup-limited.

On the other hand, most of the reported graphene photodetectors have a responsivity of less than 100 mA/W^19,21,25–29,31–33^ when operating at a low bias voltage, e.g., |*V*_b_| < 0.3 V. It is well known that, for MGM photodetectors, the responsivities are usually positively correlated with the bias voltages *V*_b_^19–21,23,25–30,35,36,43,49^ and negatively correlated with the input optical powers *P*_in_^20,35,36^. Meanwhile, it is usually desirable to be able to detect a low optical power with a low bias voltage because this helps to reduce the dark currents and suppress the shot noise. In Fig. 7, the device responsivities are shown for bias voltages of *V*_b_ = ±0.3 V unless no data are provided in the literature. Three graphene photodetectors with a responsivity of >100 mA/W have been reported recently^20,28,30^. For the photodetector reported in ref. ^28^, the responsivity is estimated to be ~150 mA/W (at 0.3 V) with *P*_in_ = 0.025 mW according to the responsivities given for the cases of *V*_b_ = 0 and 1.2 V. The other photodetector in ref. ^30^ has a responsivity of ~140 mA/W (at 0.3 V) with *P*_in_ = 0.56 mW, which is estimated from the responsivities given for the cases of *V*_b_ = 0 and 0.4 V. In ref. ^20^, the responsivities are proportional to the bias voltage and are ~375 mA/W and ~150 mA/W when operating with *V*_b_ = −0.3 V for *P*_in_ of 0.08 and 0.6 mW, respectively. For the present photodetector (Device C) operating at a low bias voltage *V*_b_ = −0.3 V, the responsivity at 1.55 μm is as high as ~0.4 A/W with *P*_in_ = 0.16 mW, which is the highest value among the results of the various reported high-speed graphene photodetectors. In addition, the tunneling photodiode in ref. ^34^ with an estimated bandwidth of 56 GHz is not included in Fig. 7, since it operates with a very large bias voltage of ~10 V while the dark current can be kept on the nA scale; therefore, it can realize a high on−off current ratio with a responsivity of 240 mA/W at *P*_in_ = 0.42 mW. However, a high bias voltage results in large power consumption and cannot be supported by low-voltage CMOS drivers. In summary, the present silicon−graphene waveguide photodetector works well with a high responsivity and a high bandwidth.

## Discussion

In this paper, we have proposed and demonstrated novel silicon−graphene hybrid plasmonic waveguide photodetectors beyond 1.55 μm, which are realized by introducing an ultrathin wide silicon ridge core region with a metal cap at the top. With this design, the light absorption in graphene is enhanced while the metal absorption loss is reduced simultaneously. This design greatly facilitates effective optical absorption in graphene over a short length. Metal−graphene−metal sandwiched electrodes have also been introduced to reduce the metal−graphene-contact resistance, which helps improve the response speed. For the fabricated photodetectors, the mechanism has been revealed from the IV characteristics operating at different gate voltages. It has been shown that the dominant mechanism for the present photodetectors is the PTE effect at zero bias voltage and the BOL or PC effect at nonzero bias voltages, which help achieve high-speed responses. For the fabricated photodetector operating at 2 μm, the measured 3 dB bandwidth is >20 GHz (which is limited by the experimental setup), while the responsivity is ~70 mA/W at V_b_ = −0.3 V for *P*_in_ = 0.28 mW. In order to verify the ultrafast photodetection capability, we have also measured the frequency responses of the present waveguide photodetector operating at 1.55 μm. It is shown that the measured 3 dB bandwidth is >40 GHz (which is still limited by the setup). Meanwhile, the measured responsivity is approximately 0.4 A/W at *V*_b_ = −0.3 V for *P*_in_ = 0.16 mW, which has some advantages over other photodetectors^19–21,25–33^. It is well known that MGM graphene photodetectors usually suffer from a low signal-to-noise ratio due to the high intrinsic large dark current. Fortunately, this issue can be alleviated partially for the present device, which has relatively high responsivities at low bias voltages. In this paper, Device A at 2 μm has an NEP of 61.7−72.7 pW/Hz^1/2^, which is slightly better than that of commercial infrared photoconductive PbSe detectors (80 pW/Hz^1/2^)^51^. For the present 2 μm waveguide photodetectors with large bandwidths, there are some important application scenarios, e.g., 2 μm optical communications^2,3^, monitoring of a 2 μm pulsed laser system in mid-infrared time resolved spectroscopy^52^, nonlinear photonics^4^, and lab-on-chip sensing^5–7^. In short, the present work paves the way for achieving high-responsivity and high-speed near/mid-infrared waveguide photodetectors on silicon, which will play an important role in various applications. In future works, more efforts should be dedicated to introduce special junction structures to reduce the dark current and further extend the operation wavelength band.

## Materials and methods

### Device fabrication

The ultrathin silicon core layer was obtained from a standard 220-nm-thick SOI wafer. A thermal oxidation process was used to obtain an ~100-nm-thick silicon top layer from a standard 220-nm-thick lightly p-doped SOI wafer. EBL and ICP processes were used for the fabrication of the silicon ridge waveguide with a silicon thickness of *h*_si_ = ~100 nm, an etching depth of *h*_et_ = ~50 nm, and a ridge width of *w*_si_ = 3 μm. A 90-nm-thick aluminum gate electrode (with an ohmic contact) was fabricated by utilizing lift-off processes. A 10-nm-thick Al_2_O_3_ layer was deposited on the SOI ridge waveguide by using an atomic-layer deposition (ALD) process. The bottom layer of the side ground electrodes was made of 15/50-nm-thick Ti/Au hybrid thin films. Then, a single-layer graphene sheet was transferred onto the chip and patterned by EBL and ICP etching processes. Finally, a 50-nm-thick Au layer was deposited and patterned to form the narrow signal electrode and the top layer of the side ground electrodes.

### Transfer process of graphene

The CVD-grown graphene was obtained from ACS material LLC (single layer, on copper foil). A 300-nm-thick film of PMMA was spin-coated on the graphene/copper film at 4000 rpm. The PMMA/graphene/copper film was floated on aqueous ammonium persulfate (60 mg/mL) to remove the copper and rinsed in deionized water. Then, the film was transferred onto the chip. The graphene-covered chip was dried, baked, soaked in acetone and rinsed with isopropanol.

### Device measurement

The responsivities of the photodetectors were characterized by using low-frequency measurements. Continuous-wave light from a fiber laser was modulated with a frequency of 0.2 kHz by a chopper and then coupled to the optical waveguide by using an on-chip grating coupler. The photocurrent was then amplified and recorded by using a preamplifier and a lock-in amplifier (see Supplementary Fig. S8a, b). The input optical power *P*_in_ was estimated according to the measured coupling efficiency of the grating coupler (~10.5 dB at 2 μm) and the power splitting ratio of the directional coupler (~1 dB at 2 μm). More details on the optical power analysis are provided in Supplementary Note 4.

## Supplementary information

Supplementary Material

## References

[1] Thomson D (2016). Roadmap on silicon photonics. J. Opt..

[2] Soref R (2015). Group IV photonics: enabling 2 µm communications. Nat. Photonics.

[3] Thomson DJ (2014). Optical detection and modulation at 2 µm−2.5 µm in silicon. Opt. Express.

[4] Lin HT (2018). Mid-infrared integrated photonics on silicon: a perspective. Nanophotonics.

[5] Lewicki R (2007). Carbon dioxide and ammonia detection using 2 μm diode laser based quartz-enhanced photoacoustic spectroscopy. Appl. Phys. B.

[6] Lavchiev VM, Jakoby B (2017). Photonics in the mid-infrared: challenges in single-chip integration and absorption sensing. IEEE J. Sel. Top. Quantum Electron..

[7] Hu T (2017). Silicon photonic platforms for mid-infrared applications. Photonics Res..

[8] Xu SQ (2019). High-speed photo detection at two-micron-wavelength: technology enablement by GeSn/Ge multiple-quantum-well photodiode on 300 mm Si substrate. Opt. Express.

[9] Ackert JJ (2015). High-speed detection at two micrometres with monolithic silicon photodiodes. Nat. Photonics.

[10] Wang RJ (2015). 2 μm wavelength range InP-based type-II quantum well photodiodes heterogeneously integrated on silicon photonic integrated circuits. Opt. Express.

[11] Xia FN (2014). Two-dimensional material nanophotonics. Nat. Photonics.

[12] Koppens FHL (2014). Photodetectors based on graphene, other two-dimensional materials and hybrid systems. Nat. Nanotechnol..

[13] Romagnoli M (2018). Graphene-based integrated photonics for next-generation datacom and telecom. Nat. Rev. Mater..

[14] Chen, X. Q. et al. Graphene hybrid structures for integrated and flexible optoelectronics. *Adv. Mater*. 10.1002/adma.201902039 (2019). 10.1002/adma.201902039 31282020

[15] Castellanos-Gomez A (2015). Black phosphorus: narrow gap, wide applications. J. Phys. Chem. Lett..

[16] Youngblood N (2015). Waveguide-integrated black phosphorus photodetector with high responsivity and low dark current. Nat. Photonics.

[17] Huang L (2019). Waveguide-integrated black phosphorus photodetector for mid-infrared applications. ACS Nano.

[18] Yin YL (2019). High-speed and high-responsivity hybrid silicon/black-phosphorus waveguide photodetectors at 2 μm. Laser Photonics Rev..

[19] Schall D (2017). Graphene photodetectors with a bandwidth> 76 GHz fabricated in a 6” wafer process line. J. Phys. D: Appl. Phys..

[20] Ma P (2019). Plasmonically enhanced graphene photodetector featuring 100 Gbit/s data reception, high responsivity, and compact size. ACS Photonics.

[21] Ding, Y. H. et al. Ultra-compact integrated graphene plasmonic photodetector with bandwidth above 110 GHz. *Nanophotonics* **9**, 317–325 (2020).

[22] Xia FN (2009). Ultrafast graphene photodetector. Nat. Nanotechnol..

[23] Gan XT (2013). Chip-integrated ultrafast graphene photodetector with high responsivity. Nat. Photonics.

[24] Pospischil A (2013). CMOS-compatible graphene photodetector covering all optical communication bands. Nat. Photonics.

[25] Youngblood N (2014). Multifunctional graphene optical modulator and photodetector integrated on silicon waveguides. Nano Lett..

[26] Schall D (2014). 50 GBit/s photodetectors based on wafer-scale graphene for integrated silicon photonic communication systems. ACS Photonics.

[27] Gao Y (2018). High-performance chemical vapor deposited graphene-on-silicon nitride waveguide photodetectors. Opt. Lett..

[28] Shiue RJ (2015). High-responsivity graphene–boron nitride photodetector and autocorrelator in a silicon photonic integrated circuit. Nano Lett..

[29] Schuler S (2016). Controlled generation of a p–n junction in a waveguide integrated graphene photodetector. Nano Lett..

[30] Schuler S (2018). Graphene photodetector integrated on a photonic crystal defect waveguide. ACS Photonics.

[31] Marconi, S. et al. Waveguide integrated CVD graphene photo-thermo-electric detector with> 40GHz bandwidth. In: *Proc. Conference on Lasers and Electro-Optics* STh4N.2 (Optical Society of America, Munich, 2019).

[32] Muench JE (2019). Waveguide-integrated, plasmonic enhanced graphene photodetectors. Nano Lett..

[33] Li TT (2018). Spatially controlled electrostatic doping in graphene p-i-n junction for hybrid silicon photodiode. npj 2D Mater. Appl..

[34] Gao Y (2019). High-speed van der Waals heterostructure tunneling photodiodes integrated on silicon nitride waveguides. Optica.

[35] Casalino M (2018). Free-space schottky graphene/silicon photodetectors operating at 2 μm. ACS Photonics.

[36] Cakmakyapan S (2018). Gold-patched graphene nano-stripes for high-responsivity and ultrafast photodetection from the visible to infrared regime. Light.: Sci. Appl..

[37] Yan J (2012). Dual-gated bilayer graphene hot-electron bolometer. Nat. Nanotechnol..

[38] Liu CH (2014). Graphene photodetectors with ultra-broadband and high responsivity at room temperature. Nat. Nanotechnol..

[39] Badioli M (2014). Phonon-mediated mid-infrared photoresponse of graphene. Nano Lett..

[40] Freitag M (2014). Substrate-sensitive mid-infrared photoresponse in Graphene. ACS Nano.

[41] Wang XM (2013). High-responsivity graphene/silicon-heterostructure waveguide photodetectors. Nat. Photonics.

[42] Cheng, Z. Z. et al. Graphene on silicon-on-sapphire waveguide photodetectors. In: *Proc. Conference on Lasers and Electro-Optics* STh1I.5 (Optical Society of America, Busan, 2015).

[43] Qu, Z. et al. Waveguide integrated graphene mid-infrared photodetector. In: *Proc. SPIE 10537, Silicon Photonics XIII* 105371N (SPIE, San Francisco, 2018).

[44] Franklin AD (2012). Double contacts for improved performance of graphene transistors. IEEE Electron. Device Lett..

[45] Koester SJ, Li M (2014). Waveguide-coupled graphene optoelectronics. IEEE J. Sel. Top. Quantum Electron..

[46] Guo JS, Wu ZW, Zhao YL (2017). Enhanced light absorption in waveguide Schottky photodetector integrated with ultrathin metal/silicide stripe. Opt. Express.

[47] Tielrooij KJ (2015). Hot-carrier photocurrent effects at graphene-metal interfaces. J. Phys.: Condens. Matter.

[48] Ma Q (2014). Competing channels for hot-electron cooling in graphene. Phys. Rev. Lett..

[49] Freitag M (2013). Photoconductivity of biased graphene. Nat. Photonics.

[50] Varykhalov A (2010). Effect of noble-metal contacts on doping and band gap of graphene. Phys. Rev. B.

[51] Thorlabs. Infrared Detectors. https://www.thorlabs.com/newgrouppage9.cfm?objectgroup_id=6479 (2019).

[52] Härkönen A (2010). Picosecond passively mode-locked GaSb-based semiconductor disk laser operating at 2 μm. Opt. Lett..

