# Supplementary material for: High-performance silicon−graphene hybrid plasmonic waveguide photodetectors beyond 1.55 μm
Source: Light Sci Appl. 2020 Feb 28;9:29. doi: 10.1038/s41377-020-0263-6 (PMC7048841; doi:10.1038/s41377-020-0263-6)
Supplement: Supplementary file 1 — Supplementary Material [file 41377_2020_263_MOESM1_ESM.docx]

**Supplementary Information**

**High-performance silicon-graphene hybrid plasmonic waveguide photodetectors beyond 1.55 μm**

Jingshu Guo^1,2^, Jiang Li^1^, Chaoyue Liu^1^, Yanlong Yin^1^, Wenhui Wang^3^, Zhenhua Ni^3^, Zhilei Fu^4^, Hui Yu^4^, Yang Xu^2,4^, Yaocheng Shi^1,2^, Yungui Ma^1^, Shiming Gao^1,2^, Liming Tong^1^ and Daoxin Dai^1,2^*

*1. State Key Laboratory for Modern Optical Instrumentation, Zhejiang Provincial Key Laboratory for Sensing Technologies, College of Optical Science and Engineering,* *International Research Center for Advanced Photonics, Zhejiang University, Zijingang Campus, Hangzhou, 310058, China.*

*2. Ningbo Research Institute, Zhejiang University, Ningbo 315100, China.*

*3. Department of Physics and Key Laboratory of MEMS of the Ministry of Education, Southeast University, Nanjing 211189, China.*

*4. College of Information Science and Electronic Engineering, Zhejiang University, Hangzhou,* Zhejiang, 310027, China.

*These authors contributed equally to this work:* Jingshu Guo, Jiang Li*.*

*Correspondence: DX Dai, State Key Laboratory for Modern Optical Instrumentation, Zhejiang Provincial Key Laboratory for Sensing Technologies, College of Optical Science and Engineering, International Research Center for Advanced Photonics, Zhejiang University, Zijingang Campus, Hangzhou, 310058, China.

*Corresponding author e-mail: dxdai@zju.edu.cn.

# **Supplementary Note 1. Characteristic analysis of the silicon-graphene hybrid plasmonic waveguide.**

For the mode analysis of the silicon-graphene hybrid plasmonic waveguide, an FEM mode-solver from COMSOL was used. In this calculation, the material refractive indices were respectively set for 2 μm and 1.55 μm as follows: n_Si_=3.451/3.476^1^, n_SiO2_=1.438/1.444^2^, n_Al2O3_=1.738/1.746^3^, and n_Au_=0.844−12.76i/0.58-9.86i^4^. Graphene is incorporated by a surface conductivity model, which has been shown working well for the graphene photonic device modeling^5,6^. Currently the surface conductivity model has been popular because it is efficient for the grid meshing in the numerical simulations^7^. The optical conductivity of graphene was calculated from the Kubo formula^8^, considering both inter- and intra-band transition terms, as shown in Fig. S1. In the simulations, we set the graphene chemical potential *μ_c_* to −0.1 eV, in which situation *V*_b_= 0, *V*_G_= *V*_Dirac_−0.15 V. Correspondingly the real parts of the optical conductivities basically are *σ*_0_= 60.8 μS for both 2 μm and 1.55 μm (see Fig. S1).

When the voltages *V*_G_ and *V*_b_ vary, the chemical potential and the optical conductivity distributions for the graphene sheet change, while the mode field distributions change very slightly. It is noted that the light absorption of graphene is mainly decided by the real part (rather than the imaginary part) of its optical conductivity, and the light absorption mainly happens in the area close to the signal-electrode at the middle. Therefore, the real part of the optical conductivity of the graphene in the area close to the signal electrode is the key factor. Due to the pinning effect^9^, the chemical potential *μ_c_* of the graphene sheet underneath the signal-electrode (gold) is usually fixed to −0.1 eV according to the result in ref. 10. For the graphene sheet around the signal-electrode (gold), the chemical potential *μ_c_* deviates slightly from −0.1 eV even for varied voltages *V*_G_ and *V*_b_. Meanwhile, the real part of the graphene optical conductivity varies very slightly for both wavelengths of 2 μm and 1.55 μm when |*μ_c_*|<0.2 eV, as shown in Fig. S1a, b. Therefore, the simulation results in Fig. S1a, b for the case of *V*_b_=0, *V*_G_=*V*_Dirac_−0.15 V are still valid even for varied voltages *V*_b_ and *V*_G_.


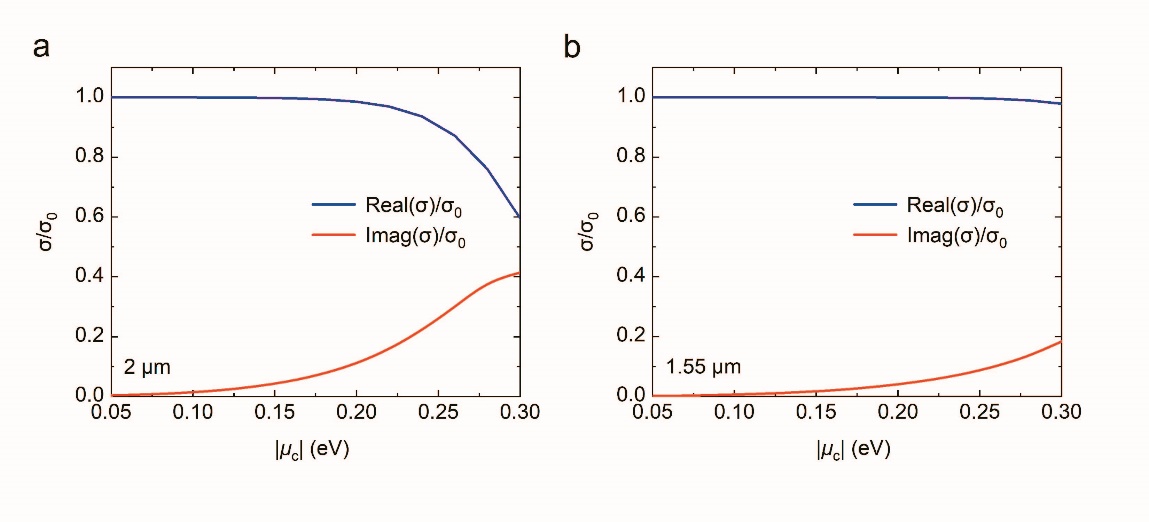


**Fig. S1** **The graphene optical conductivity versus the chemical potential. a** Wavelength is 2 μm; **b** Wavelength is 1.55 μm.

As described in the main text, for a given propagation length of *L*, the graphene light absorptance can be expressed by $\eta\left( L \right)= \eta_{g}\left( 1-e^{-\alpha_{e}L} \right)$, in which $\eta_{g}$ is the graphene absorption ratio, and $\alpha_{e}$ is the mode absorption coefficients in μm^-1^. One has $\alpha_{e}=\alpha/4.34$, where *α* is the waveguide loss in dB/μm. The graphene absorptance is calculated by^1^

$\eta\left( L \right)=\frac{\int_{0}^{L} \int A_{g}(l)e^{-\alpha_{e}z}dl\mathrm{dz}}{P_{0}}=\frac{1}{\alpha_{e}}(1-e^{-\alpha_{e}L})\frac{\int A_{g}(l)dl}{P_{0}}$ , (Eq. S1)

where *P*_0_ is the input mode power, *l* is the coordinate of the line integral along the graphene surface in the *xy* plane, *A*_g_(*l*) is the graphene light absorption intensity. One has $A_{g}(l)=\frac{1}{2}\mathrm{Real}(\sigma_{g})\left| \vec{E}_{t}(l) \right|^{2}$(W/m^2^), where Real(*σ*_g_) is the real part of the graphene conductivity, $\vec{E_{t}}$ is the transverse component of the electric fields along the graphene surface of the launched waveguide mode (at *z* =0). Similarly, the metal absorptance is calculated by^11^

$\eta_{m}\left( L \right)=\frac{\int_{0}^{L} \iint A_{m}{(x,y)e}^{-\alpha_{e}z}dxdydz}{P_{0}}=\frac{1}{\alpha_{e}}(1-e^{-\alpha_{e}L})\frac{\iint A_{m}(x,y)dxdy}{P_{0}}$ . (Eq. S2)

Here the integral area of the *xy*-plane surface integral is in the metal area, and *A*_m_(*x*, *y*) is the metal absorption intensity given by $A_{m}(x,y)=\frac{1}{2}\omega\cdot\mathrm{Imag}(\varepsilon_{m})\left| \vec{E}(x,y) \right|^{2}$(W/m^3^), where *ω* is the angular optical frequency, Imag(*ε*_m_) is the imaginary part of the metal permittivity,$\vec{E}$ is the electric fields in the metal area. One has $\alpha_{e}=\frac{\int A_{g}(l)dl}{P_{0}}+\frac{\iint A_{m}(x,y)dxdy}{P_{0}}$ according to Eqs. (S1) and (S2). The graphene absorption coefficient α_eg_ and the metal absorption coefficient α_em_ are given as (in μm^-1^)

$\alpha_{\mathrm{eg}}=\frac{\int A_{g}(l)dl}{P_{0}}$, (Eq. S3)

$\alpha_{\mathrm{em}}=\frac{\iint A_{m}(x,y)dxdy}{P_{0}}$. (Eq. S4)

Then one has $\alpha_{e}=\alpha_{\mathrm{eg}}+\alpha_{\mathrm{em}}$, and the graphene absorption ratio is given by $\eta_{g}=\frac{\eta\left( L \right)}{\eta\left( L \right)+\eta_{m}\left( L \right)}=\frac{\alpha_{\mathrm{eg}}}{\alpha_{\mathrm{eg}}+\alpha_{\mathrm{em}}}$. Since ${\alpha_{g}=4.34\alpha}_{\mathrm{eg}}$ and ${\alpha_{m}=4.34\alpha}_{\mathrm{em}}$, one has $\eta_{g}=\frac{\alpha_{g}}{\alpha_{g}+\alpha_{m}}$. With these formulas, the absorption coefficients in dB/μm (*α*_g_, *α*_m_) and the graphene absorption ratio *η*_g_ can be calculated as the waveguide dimensions varies. The calculation results for the quasi-TE_0_ mode of the silicon-graphene hybrid waveguide operating at 2 μm are given in Fig. 2 in the maintext. We also give an analysis for the same waveguide operating at 1.55 μm, as shown in Fig. S2. It can be seen the waveguide designed for the wavelength-band of 2 μm also works well for the wavelength-band of 1.55 μm. Figure S2a shows the dependence of the graphene absorption ratio *η*_g_ and the absorption coefficients (*α*_g_, *α*_m_) on the width *w*_m_ and the height *h*_m_ of the metal strip when *w*_si_= 3 μm and *h*_si_= 100 nm. When the metal strip becomes wider, the graphene absorption coefficient *α*_g_ is higher and the graphene ratio *η*_g_ becomes lower. On the other hand, the graphene absorption coefficient *α*_g_ is lower and the graphene ratio *η*_g_ becomes higher when choosing a thicker metal strip.

The waveguide structure with (*w*_m_, *h*_m_)=(200 nm, 50 nm) designed for the 2 μm wavelength- band also works for the 1.55 μm wavelength-band. In this case, one has (*α*_g_, *α*_m_) = (0.295, 0.181) dB/μm, and *η*_g_=62.0%. Supplementary Figure S2b shows the transversal electric field distribution of the designed waveguide operating at 1.55 μm. The electric field components $\sqrt{\left| \vec{E_{x}} \right|^{2}+\left| \vec{E_{z}} \right|^{2}}$ along the graphene layer at the metal corners reach up to 1.22×10^7^ V/m for 1 mW input power. As shown in Fig. S2c, the graphene absorptance of this designed waveguide is 54.3% when choosing the device length as short as 20 μm. When the metal width has some deviation to be e.g. *w*_m_= 300 nm, the graphene absorptance is close to the saturated value of 42.1% for the length *L* as short as 10 μm, which is due to the high absorption of the metal strip.


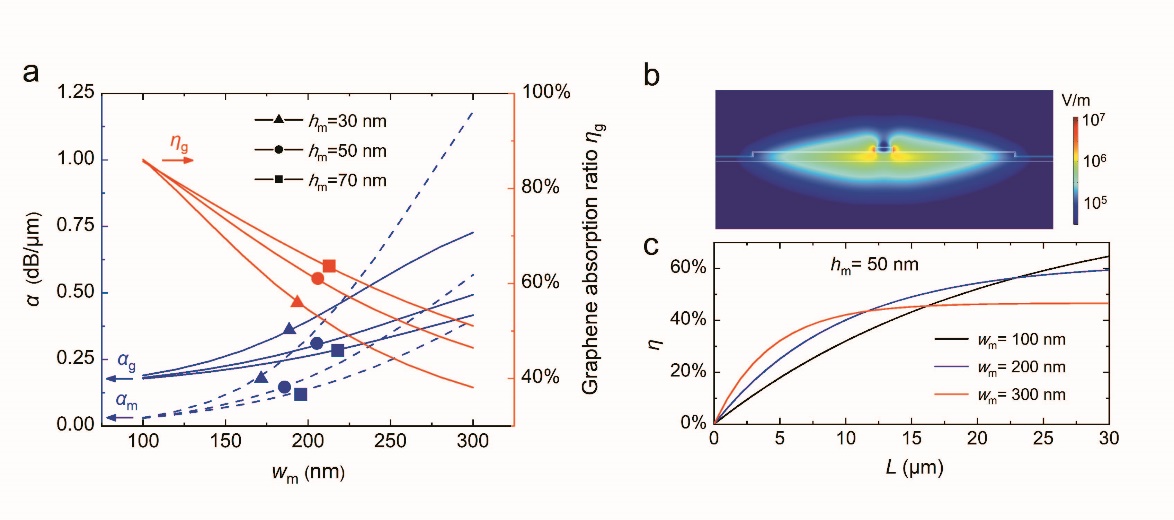


**Fig. S2** **Mode properties of the present silicon-graphene hybrid plasmonic waveguide when operating at λ=1.55 μm**. **a** Calculated absorption coefficients (*α*_g_, *α*_m­_)*,* and the graphene absorption ratio *η*_g_ as the metal-strip width *w*_m_ varies for the cases with different metal heights *h*_m_. Here *w*_si_=3 μm, and *h*_si_= 100 nm; **b** The electric field component $\sqrt{\left| \vec{E_{x}} \right|^{2}+\left| \vec{E_{z}} \right|^{2}}$ of the quasi-TE mode for the optimized silicon-graphene hybrid plasmonic waveguide (@ 1.55 μm); **c** Calculated graphene absorptance *η* as the propagation length *L* varies for the cases with different metal-strip widths *w*_m_ = 100, 200, and 300 nm. Here *h*_m_= 50 nm, *w*_si_= 3 μm, and *h*_si_= 100 nm.

# **Supplementary Note 2. Device parameter characterization by capacitance model.**

For the fabricated devices, the I-V characteristics were characterized under varied gate voltages and a fixed low bias voltage supplied by two sourcemeters (Keithley 2401) in the dark case (i.e., the input optical power *P*_in_=0). From the measured I-V curves, the total resistance *R*_tot_ can be achieved easily. As it is well known, the total resistance for the photodetector based on a metal-graphene-metal structure is given by $R_{\mathrm{tot}}=R_{c}+0.5\frac{W_{g}}{L_{g}}\sigma^{-1}$, where *R*_c_ is the total contact resistance of the graphene-metal ohmic contacts^12^, *W*_g_ and *L*_g_ are respectively the width and the length of the graphene channel between the signal- and ground-electrodes, *σ* is the graphene conductivity. The graphene conductivity is given as $\sigma=\sqrt{\sigma_{\min}^{2}+\left[ \mu C_{G}{(V}_{G}-V_{\mathrm{Dirac}}) \right]^{2}}$, where *σ*_min_ is the minimal conductivity, *μ* is the graphene mobility, *V*_Dirac_ is the Dirac gate voltage, *C_G_* is the gate capacitance given by $C_{G}={\varepsilon_{0}\varepsilon_{\mathrm{Al}_{2}O_{3}}}/{h_{\mathrm{Al}_{2}O_{3}}=8\times{10}^{-3}F/{m^{2}}}$. Therefore, the contact resistance *R*_c_ and the graphene properties (*σ*_min_, *μ*, and *V*_Dirac_) can be obtained by fitting the measured data for the total resistances *R*_tot_. For example, for Device A with *L*_g_= 50 μm and *W*_g_= 2.8 μm, the measured total resistance *R*_tot_ is shown in Fig. S3a. It can be seen that the total resistance becomes the maximal at *V*_G_= 3.2 V, which corresponds to the Dirac voltage. The other fitted parameters are *R*_c_= 45 Ω, *σ*_min_= 0.206 mS, and *μ*= 522 cm^2^/V·s. For Device A, the minimum of the total resistance *R*_tot_ is ~60 Ω when choosing *V*_G_= −2 V, while the normalized contact resistance *R*_c_·*L*_g_ is about 2250 Ω·μm.

The measured data for another two devices (Devices B and C) are also given, as shown in Fig. S3b. For these two devices, the graphene is highly doped, and their Dirac voltages *V*_Dirac_ are larger than 4 V, and thus a low gate voltage (e.g., < 4V) does not introduce significant influence on the device resistances. Similarly, the contact resistance *R*_c_ and the graphene properties (*σ*_min_, *μ*, and *V*_Dirac_) for Devices B and C can also be obtained by fitting the measured data for the total resistances *R*_tot_. For Device B with *L*_g_=50 μm and *W*_g_=2.8 μm and Device C with *L*_g_=20 μm and *W*_g_=2.2 μm (operating at 1.55 μm), their contact resistances *R*_c_ are estimated as ~60 Ω and ~104 Ω, respectively. It can be seen that Devices A, B, and C have normalized contact resistances in the range of 2000~3000 Ω·μm. In our devices, the contact resistance *R*_c_ depends on both the contact-resistances for the signal-electrode at the middle as well as the ground-electrodes at the sides. One should notice that the contact-resistances for the signal-electrode might be the dominant one because the signal-electrode is much narrower width than the ground-electrodes.


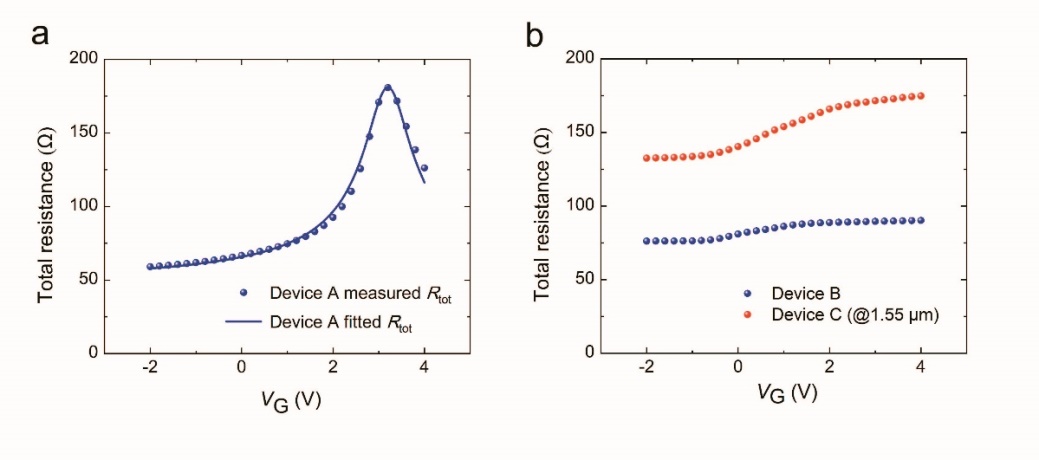


**Fig. S3** **Total device resistance *R*_tot_.** **a** Measured and fitted *R*_tot_ of Device A; **b** Measured *R*_tot_ for Devices B and C.

# **Supplementary Note 3. Device parameter characterization by equivalent circuit model.**

As it is well known, for photodetectors, the bandwidth is mainly determined by the photo-response time and the electric-circuit response time. It has been early found that the photo-response can be very fast (~2 ps) in the graphene with low or high doping, due to the short lifetime of the photogenerated carriers^13^. The photoconductive photo-response limited bandwidth can be even over 260 GHz^13^. Besides, the BOL effect in graphene can also be fast, even though the bulk semiconductor bolometers typically work in kHz. According to the recent works on the dynamic thermal response in graphene, the thermal response time is ~100 ps at low temperature of 10 K^14^ and only several picoseconds at room temperature^15^. There have been several graphene bolometric photodetectors reported with ultra-large bandwidth, e.g., 76 GHz^16^, and 110 GHz^17^. As it can be seen, the ultrafast thermal response makes it possible to realize graphene bolometric photodetectors with thermal-relaxation-limited bandwidths much larger than 100 GHz. Therefore, for the present graphene photodetectors, the bandwidths may be electric-circuit-limited for both two operation modes (i.e., the BOL effect and the PC effect), and thus the high frequency responses in ~GHz range are mainly focused in this work.

Since the 3 dB-bandwidth of the present photodetectors is beyond the setup-limit (with the maximal frequency *f*_max_), here we establish an equivalent circuit model with the parameters extracted from the measured S_11_, so that one can estimate the electric-circuit-limited 3 dB- bandwidth from the calculated electric-circuit frequency response S_21_^EC^. Figure S4a, b shows the equivalent circuits, where *I*_ph_(ω) is the photocurrent source, *C*_pad_ is the pad capacitance, *C*_g_ and *R*_g_ are respectively the capacitance and the resistance corresponding to the graphene area, *C*_oxc_ and *C*_oxs_ are respectively the capacitances corresponding to the Al_2_O_3_ layer at the middle and the Al_2_O_3_ layer at the sides, *R*_Si_ is the silicon resistance.

According the structural symmetry of the device, this equivalent circuit model can further be simplified, as shown in Fig. S4a, where *C*_tot_=2(*C*_g_ +*C*_pad_), $C_{\mathrm{ox}}=\frac{{2C}_{\mathrm{oxc}}C_{\mathrm{oxs}}}{C_{\mathrm{oxs}}+C_{\mathrm{oxc}}}$, and *R*_tot_= 0.5 *R*_g_. Then the theoretical frequency-dependent impedance *Z*_in_^T^ of the photodetector is given by

$Z_{in}^{\text{T}}(\omega)=\frac{\frac{R_{tot}}{j\omega R_{tot}C_{tot}+1}\left(\frac{1}{2}R_{si}+\frac{1}{j\omega C_{ox}}\right)}{\frac{R_{tot}}{j\omega R_{tot}C_{tot}+1}+\frac{1}{2}R_{si}+\frac{1}{j\omega C_{ox}}}$. (Eq. S5)

Meanwhile, the reflection coefficient S_11_ in the setup-limited frequency range (*f*<*f*_max_) was measured by using a vector network analyzer (VNA) (shown in Fig. S4a) with the same gate voltage as that applied for the measurement of the corresponding S_21_ response. Then the measured frequency-dependent impedance can be given by *Z*_in_^M^ (ω)=*Z*_0_[1+*S*_11_(ω)]/[1−*S*_11_(ω)], where *Z*_0_=50 Ω. All these parameters (*R*_tot_, *C*_tot_, *R*_si_, and *C*_ox_) can be extracted by fitting *Z*_in_^M^ and *Z*_in_^T^ in the frequency range of *f*<*f*_max_. Finally, one can calculate the electric circuit frequency response S_21_^EC^ theoretically in the frequency range beyond the setup-limited frequency *f*_max_ according to the established equivalent circuit model. In Fig. S4b, the simplified equivalent circuit for S_21_^EC^ evaluation is given, where *R*_L_ is the standard 50 Ω load resistance and *I*_L_(ω) is the signal photocurrent. S_21_^EC^ is given by

$S_{21}^{EC}=\left|\frac{I_{L}(\omega)}{I_{source}(\omega)}\right|^{2}=\left|\frac{Z_{in}}{Z_{in}+R_{L}}\right|^{2}$. (Eq. S6)


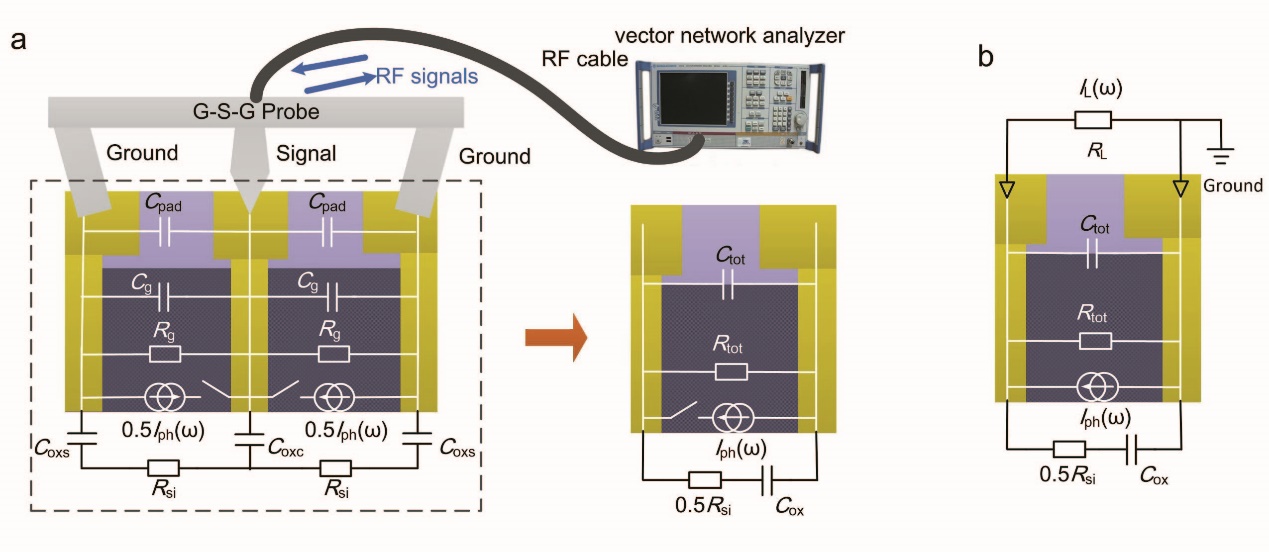


**Fig. S4** **Schematic diagrams of equivalent circuit model. a** Detailed and simplified equivalent circuits with the S_11_ measurement setup. **b** Simplified equivalent circuit for electric circuit frequency response S_21_^EC^ evaluation.

Supplementary Figure S5a shows the measured impedances *Z*_in_^M^ and fitting impedances *Z*_in_^T^ of the photodetectors when operating at different conditions. The parameters for the RC elements in the equivalent circuit were extracted and shown in Table S1. The fitting results for the total resistance given in Table S1 are very similar to the static measurement results (e.g., for Device A, *R*_tot_=~98 Ω @*V*_G_=2.1 V, ~150 Ω @ *V*_G_=3.5 V), which indicates the established equivalent circuit works well for the present photodetectors.


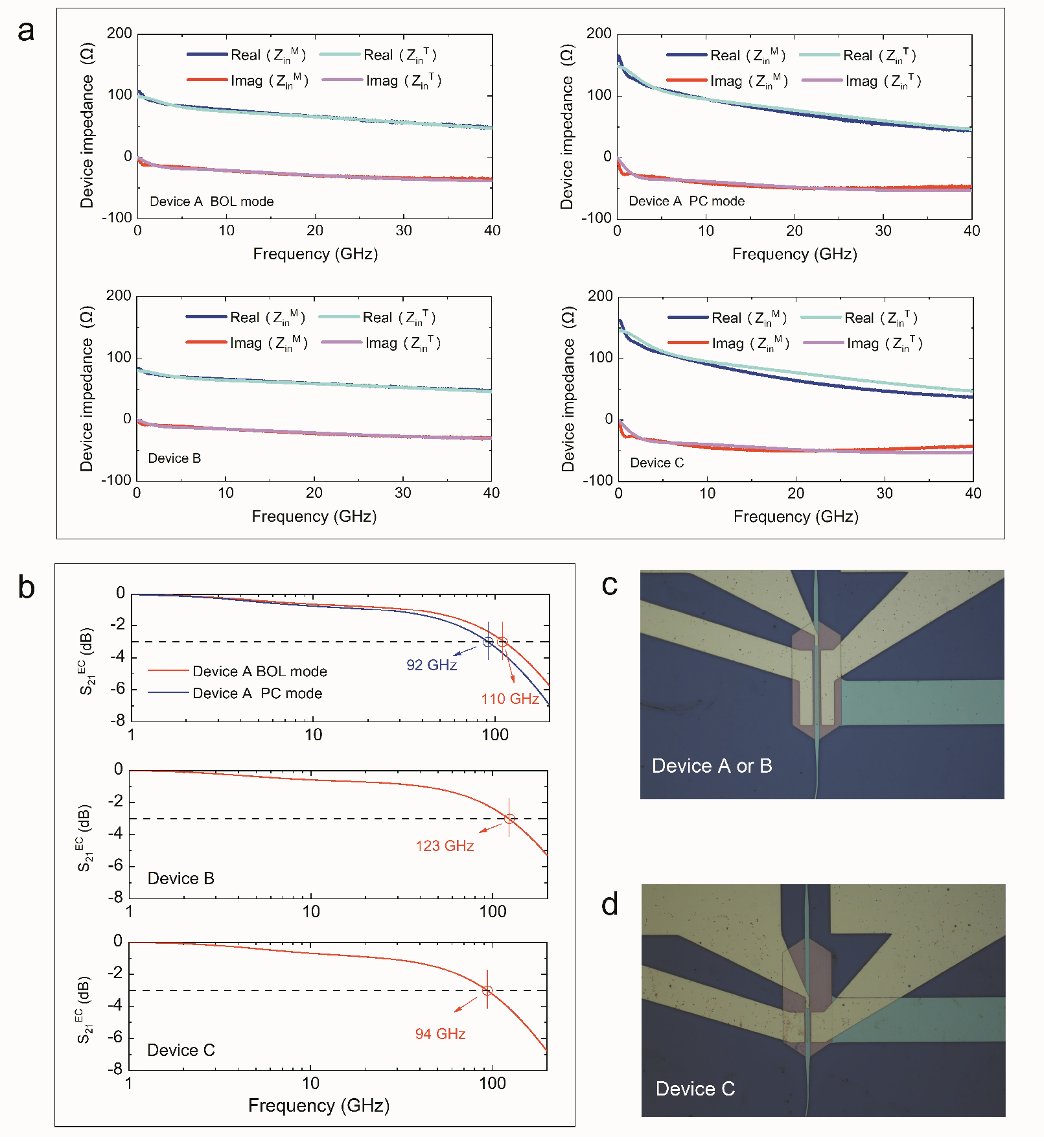


**Fig. S5** **Results of the equivalent circuit model. a** The measured impedances *Z*_in_^M^ and fitting impedances *Z*_in_^T^ given by real parts and imaginary parts for Device A at the BOL mode with *V*_G_=2.1V, Device A at the PC mode with *V*_G_=3.4V, Device B at the BOL mode with *V*_G_=2.9V, and Device C at the BOL mode with *V*_G_=2.8V; **b** The calculated electric circuit frequency response S_21_^EC^; **c** Structure of Device A or B; **d** Structure of Device C.

According the established equivalent circuit model, the calculated electric circuit frequency responses S_21_^EC^ are shown in Fig. S5b, and the estimated 3 dB-bandwidths are listed in Table S1. For Device A, the estimated 3 dB-bandwidths are about 110 GHz and 92 GHz when operating with the BOL effect and the PC effect, respectively. The difference is mainly due to their different total resistances *R*_tot_. As shown in Table S1, the total resistance *R*_tot_ (98.6 Ω) under the BOL mode is lower than that (148.5 Ω) under the PC mode, which is due to the higher doping level in graphene. Accordingly, the estimated 3dB-bandwidth for Devices B is about 123 GHz, which is higher than that for Device C (94 GHz). Therefore, all these devices have potentials to realize ~100 GHz operations. According to the equivalent circuit model, the 3 dB-bandwidths of the present photodetectors are mainly determined by the total resistance *R*_tot_ as well as the total capacitance *C*_tot_. All the parameters are analyzed as below.

1. About the total resistance *R*_tot_. For Devices B and C, the lengths *L*_g_ of their graphene sheets are respectively 50 μm and 20 μm, while the electrode spacings for them are 2.8 μm and 2.2 μm. As a result, Device C has a higher total resistance *R*_tot_ than Device B.
2. About the total capacitance *C*_tot_. The total capacitance *C*_tot_ consists of two parts. One is the capacitance 2·*C*_g_ for the graphene areas and the other one is the 2·*C*_pad_ for the metal pads/connectors. Here two-dimensional numerical simulations (using COMSOL Electrostatics Interface) were performed to evaluate the capacitances 2·*C*_g_ and 2·*C*_pad_. The simulation shows that the calculated normalized capacitances for the graphene areas of Devices B and C are about 32.1 pF/m and 34.5 pF/m. Accordingly, one has 2·*C*_g_=1.6 fF and 0.69 fF for Devices B and C regarding the length *L*_g_=50 μm and 20 μm, respectively. The capacitance 2·*C*_pad_ consists of two parts contributed by the rectangular metal-pads and the metal-connectors. The capacitances contributed by the metal-connectors for all the devices are similar since their metal-connectors have similar shapes and sizes. For the present devices, the calculated results is ~6 fF capacitances. For the part contributed by the rectangular metal-pads, the calculated normalized capacitance is about 165 pF/m, which gives a capacitance of 26.4 fF for the 160 μm-long rectangular metal-pad accordingly. As a result, the capacitance 2·*C*_pad_ contributed by the rectangular metal-pads and the metal-connectors is about 32 fF for all the devices, which is much higher than the graphene capacitance 2·*C*_g_. Accordingly, all the devices with different lengths *L*_g_ have similar capacitances *C*_tot_ (~32 fF), which are consist with the fitted parameters (~40 fF) given in Table S1.
3. About the silicon resistance *R*_si_. The resistance *R*_si_ depends on the device dimensions and the electrode layout. Even though Device C is shorter than Devices A and B, the resistances *R*_si_ for these three devices are similar. One of the reasons is that Device C has narrower electrode-spacing than Devices A and B. Furthermore, the area of the signal-electrode sitting on the silicon layer for Device C is larger than that for Devices A and B, as shown in Fig. S5c, d.
4. About the Al_2_O_3_-layer capacitance *C*_ox_. According to the device structure, the Al_2_O_3_-layer capacitance *C*_ox_ is proportional to the overlap area between the electrode and the silicon core-layer. In Fig. S5c, d, the detailed structures of Devices A & B and Device C are given by optical microscope pictures. For the present device, there are two parts contributed to the capacitance *C*_ox_. One is for the signal-electrode at the middle and the other one is for the ground-electrodes at both sides. For Devices A and B (see Fig. S5c), the overlap area of the signal-electrode is much smaller than that of the ground-electrodes, and thus the capacitance *C*_oxc_ corresponding to the signal-electrode is much smaller than the capacitance *C*_oxs_ corresponding to the ground-electrodes, i.e., *C*_oxc_ << *C*_oxs_. As a result, the capacitance *C*_oxc_ is the dominant one. In contrast, for Device C, the area of the central electrode is actually similar to the ground-electrodes because there is a relatively large metal pad at the rear (see Fig. S5d). On the other hand, the length of the ground-electrodes for Device C is shorter than that for Devices A and B. As a result, these devices have similar capacitances *C*_ox_ while Device C is the smallest one, as shown in Table S1.

**Table S1. The parameters for the equivalent circuit extracted from the measured S_11_ and the estimated electric-circuit-limited 3dB-bandwdith BW_RC-3dB_.**

| Devices | Mechanism | *R*_tot_  (Ω) | 0.5·*R*_si_  (Ω) | *C*_tot_  (fF) | *C*_ox_  (fF) | BW_RC-3dB_  (GHz) | *L*_g_  (μm) | *W*_g_  (μm) | *λ*  (μm) |
| --- | --- | --- | --- | --- | --- | --- | --- | --- | --- |
| A | BOL effect | 98.6 | 370.8 | 39.7 | 95.2 | 110 | 50 | 2.8 | 2 |
|  | PC effect | 148.5 | 362.2 | 41.4 | 97.8 | 92 |  |  |  |
| B | BOL effect | 79.8 | 375.8 | 38.8 | 97.1 | 123 |  |  |  |
| C | BOL effect | 145.6 | 370.1 | 40.5 | 79.2 | 94 | 20 | 2.2 | 1.55 |

# **Supplementary Note 4. Characterization of the optical transmissions.**

As shown in Fig. S6a, light was coupled from an optical fiber to the waveguide photodetector by using a grating coupler. Here a directional coupler (DC) with a power splitting ratio of 90%:10% was inserted before the photodetector to make the fiber-alignment convenient. When operating at 2 μm, the fabricated grating coupler has a coupling loss of ~10.5 dB and the fabricated DC has a loss of ~1 dB. In contrast, when operating at 1.55 μm, the fabricated grating coupler has a coupling loss of about 8.5 dB and the fabricated DC has a loss of 1~2 dB (due to some unexpected fabrication variations). The input optical power *P*_in_ can be calculated by subtracting the grating coupler loss and the DC loss from the optical power *P*_inchip_ launched from the fiber.


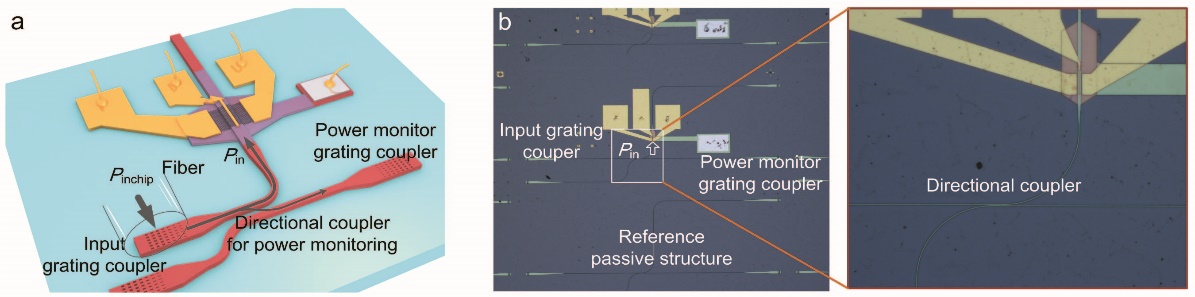


**Fig. S6** **Fabricated photonic integrated circuits.** **a** Schematic configuration; **b** microscopy pictures.

**Supplementary Note 5. Field effect modeling.**

In this section, *l* is the one-dimensional coordinate to define the location in the graphene channel, the two end-positions of graphene conducting signal electrode and ground electrode are respectively defined as *l*=0 and *l*=*W*_g_ (*W*_g_ is the graphene channel width, see Supplementary Note 2). The chemical potential *μ_c_*(*l*) denotes the difference between the Fermi level *E*_F_(*l*) and the Dirac-point energy *Φ*(*l*), i.e.,^18^:

$E_{F}(l)=\Phi(l)+\mu_{c}(l)$. (Eq. S7)

Usually *E*_F_(*l*) is assumed to be varying linearly along the graphene channel^18^, i.e.,

$E_{F}\left( l \right)=V_{b}\left( l \right)e=(1-\frac{l}{W_{g}})V_{b}e$ (0 ≤ *l* ≤ *W*_g_), (Eq. S8)

where *V*_b_ is the bias voltage, and *V*_b_(*l*) is the potential in channel, *e* is unit negative charge. Obviously, one has $E_{F}\left( l \right)=V_{b}e\left( l<0 \right) or 0(l>W_{g})$. In this work, the width of the transition regions between the pinning regions and the fully gate-controllable region is set to 0.3 μm^23^. In the graphene region unaffected by the metal pinning effect (i.e., 0.3 μm ≤ *l* ≤ *W*_g_−0.3 μm), the electrostatic-doping induced net charge-density *n*(*l*) can be calculated by^19,20^

$n(l)=\frac{C_{G}[V_{G}-V_{b}(l)-V_{\mathrm{Dirac}}]}{|e|}$, (Eq. S9)

where *C*_G_ is the capacitance. The net charge density *n*(*l*) is related to the chemical potential *μ_c_*(*l*) by^21^

$n(l)=n_{e}-n_{h}=\frac{2}{\pi(\hbar v_{F})^{2}}\int_{0}^{\infty} (\frac{E}{e^{\frac{E-\mu_{c}(l)}{k_{B}T}}+1}-\frac{E}{e^{\frac{E+\mu_{c}(l)}{k_{B}T}}+1})dE$, (Eq. S10)

where *n*_e_ is the electron density, *n*_h_ is the hole density, and *v*_F_ is the graphene Fermi velocity (10^6^ m/s here^22^). According to Eq. (S10), the chemical potential *μ*_c_(*l*) can be calculated.

For the graphene underneath the Au electrodes (*l* ＜0 or l＞ *W*_g_), the chemical potential was pinned to −0.1eV (lightly p-doing)^10^, i.e., $\mu_{c}\left( l \right)=-0.1 eV(l<0 or l>W_{g})$, which indicates that the Fermi level is 0.1 eV lower than Dirac point. The chemical potential *μ*_c_(*l*) in the transition regions (0 ≤ *l* ≤ 0.3 μm, *W_g_−*0.3 μm ≤ *l* ≤ *W*_g_) is assumed to be varying linearly with the position *l*. With the distributions of *E*_F_(*l*) and *μ_c_*(*l*), the Dirac-point energy *Φ*(*l*) can be calculated by Eq. S7. The calculation results are shown in Fig. 3c-f of the main text.

# **Supplementary Note 6. The PTE photocurrent modeling.**

The PTE photocurrent *I*_PTE_ is evaluated by $I_{\mathrm{PTE}}=\frac{\overline{V}_{\mathrm{PTE}}}{R_{\mathrm{tot}}}$, where $\bar{V}_{\mathrm{PTE}}$ is the average PTE photovoltage, *R*_tot_ is the total resistance. The total resistance *R*_tot_ was taken from the measured IV curves (see Supplementary Note 2). The average PTE photovoltage $\bar{V}_{\mathrm{PTE}}$ was calculated with the formula $\bar{V}_{\mathrm{PTE}}=-\int S(l)\frac{d\overline{T}_{e}(l)}{dl}dl$, where *l* is the lateral position along the graphene sheet, *S*(*l*) is the Seebeck coefficient and $\frac{d\overline{T}_{e}(l)}{dl}$ is the optically-induced electron temperature gradient. For the lateral positions at the corners of the signal electrode and the right ground electrode, one has *l*=0 and 2.8 μm for Device A, respectively. The Seebeck coefficient S is expressed as $s(\mu_{c})=-\frac{\pi^{2}k_{B}^{2}T}{3e}\frac{1}{\sigma}\frac{d\sigma}{d\mu_{c}}$. The average electron temperature is given by $\overline{T}_{e}(l)=\frac{\int_{0}^{L} T_{e}(l,z)dz}{L}$, where *T*_e_ (*l*, *z*) is the electron temperature distribution obtained from the following heat equation

$-\nabla\cdot(\kappa\nabla T_{\text{e}})+\frac{\kappa}{\xi^{2}}(T_{\text{e}}-T_{0})=P(l,\text{z})$. (Eq. S11)

In Eq. S11, *T*_0_=300 K, the cooling length *ζ* is chosen as *ζ*=1 μm in graphene^24^, and the electronic thermal conductivity *κ* is given by Wiedeman-Franz relation $\kappa=\frac{\pi^{2}k_{B}^{2}T\sigma}{3e^{2}}=\text{2.44}\times\text{1}\text{0}^{-8}T\sigma[W/K]$. The optical power absorption density in Eq. S11 is given by $P\left( l,z \right)=P_{\mathrm{in}}\frac{A_{g}(l)}{P_{0}}{10}^{-0.1\alpha z}$, where *P*_in_ is the input power, and $\frac{A_{g}(l)}{P_{0}}$ is the normalized graphene absorption density in unit of m^-2^ (see Supplementary Note 1) as shown in Fig. S7a. One can see that most light absorption occurs near the interface between the graphene sheet and the signal electrode. Despite that the distributions of Seebeck coefficient and the temperature are horizontally symmetric, the PTE-induced photocurrents of the left and right half-parts accumulate due to the G-S-G electrode configuration. It is noted that the light absorption distributions are not sensitive to *V*_G_ and *V*_b_ (−0.3~0.3V). Heat dissipation happens via the metallic contacts due to the heat-sink effect for the graphene underneath the metal electrodes^25^, in which case the cooling length is reduced compared to the case for the pure graphene (without metals). Here we choose the cooling length as *ζ*_m_= 0.1 μm as an example, corresponding the case with strong heat-sink effect. In this case, the coefficient *κ*/*ζ*^2^ is 100 times higher than that for the case of ignoring the heat-sink effect and correspondingly the total power dissipation becomes 100 times higher. The calculated average electron temperature increment ($\bar{T}_{e}-T_{0}$) when *V*_G_=*V*_Dirac_ is shown in Fig. S7a. One can see that the metal heat-sink effect introduces some degradation of the electron heating, while $[\bar{T}_{e}(l=0)-T_{0}$] does not decrease to zero. The maximum value is achieved at the position close to the signal-electrode, i.e., the optically-induced electron temperature gradient $\frac{d\overline{T}_{e}(l)}{dl}$ is negative for most part of the graphene sheet. Supplementary Figure S7b shows the energy-band diagrams at the zero bias for the cases with different gate voltages *V*_G_. For the graphene sheet, there are two transition regions and a fully gate-controllable region. When *V*_b_=0, the working mechanism is possibly dominated by the PTE effect or the PV effect. If the mechanism is dominated by the PV effect, the photocurrent mainly depends on the build-in electric field d*Φ*/d*l* in the transition region close to the central metal strip. Since the build-in electric field changes monotonically as the gate voltage *V*_G_ increases, as shown in Fig. S7b, the photocurrent should change monotonically as the gate voltage *V*_G_ increases, which however does not agree with the experimental result shown in Fig. 3b in the maintext. As a conclusion, the mechanism is not dominated by the PV effect.

Instead, the dominant mechanism is likely to be the PTE effect. Supplementary Figure S7c shows the calculated PTE photo-current *I*_PTE_ as the bias voltage *V*_b_ and the gate voltage *V*_G_ varies. From this figure, one sees that the photocurrent *I*_PTE_ is highly dependent on the gate voltage. The zero point for the photocurrent *I*_PTE_(V_G_) shifts as the bias voltage *V*_b_ varies. The photocurrent *I*_PTE_ is not sensitive to the bias voltage for fixed V_G_ unless |*V*_G_−*V*_Dirac_|<0.5V. Supplementary Figure S7d shows the calculated photocurrent *I*_PTE_ at zero bias as *V*_G_ varies, which matches very well with the experimental result shown in Fig. 3b in the maintext. Therefore, it is verified that the dominant mechanism for the present photodetectors is the PTE effect when operating at zero bias.


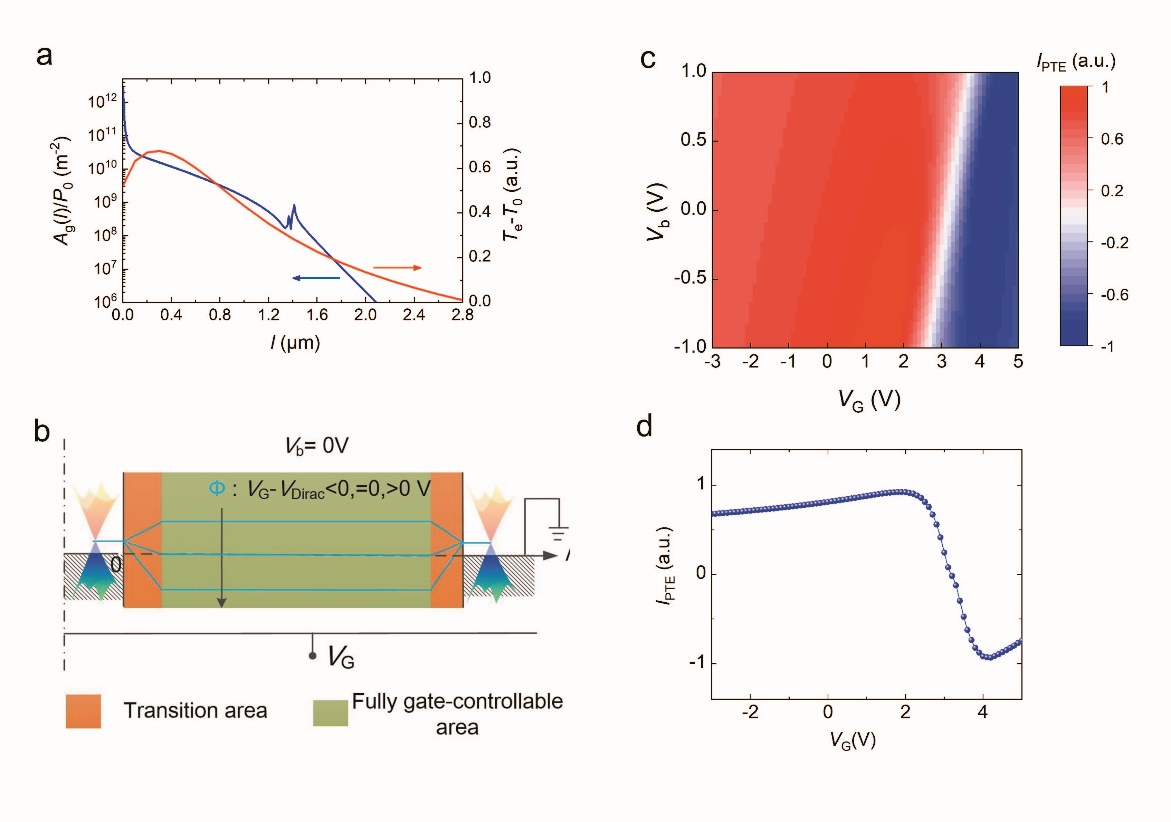


**Fig. S7** **The PTE photo-current modeling of Device A.** **a** Normalized graphene absorption density *P*(*l*) and average electron temperature increment ($\bar{T}_{e}-T_{0}$) with *V*_G_=*V*_Dirac_; **b** Energy-band diagram of the silicon-graphene photodetector with *V*_b_=0; **c** PTE photo-current as the voltages *V*_b_ and *V*_G_ varies; **d** PTE photo-current versus *V*_G_ when *V*_b_=0.

# **Supplementary Note 7. Measurement setups.**

Supplementary Figure 8a shows the low-frequency measurement setups for the devices when operating at 2 μm. Here the CW light from the 2 μm fiber laser was modulated by a 0.2 kHz chopper. The modulated light was coupled to the chip through the input grating coupler for TE polarization. The polarization controller was used before light enters the chip. The bias voltage and the gate voltage were applied by Sourcemeters (Keithley 2401) and a pre-amplifier (SR570), respectively. The electrical signal was received by using a lock-in amplifier (SR830) with help of the reference clock signal from the chopper. The photocurrent from the lock-in amplifier was used to evaluate the responsivity. For the devices operating at 1.55 μm, light from the tunable laser (HP 8163A) was internally modulated with a frequency of 1 kHz, as shown in Fig. S8b.


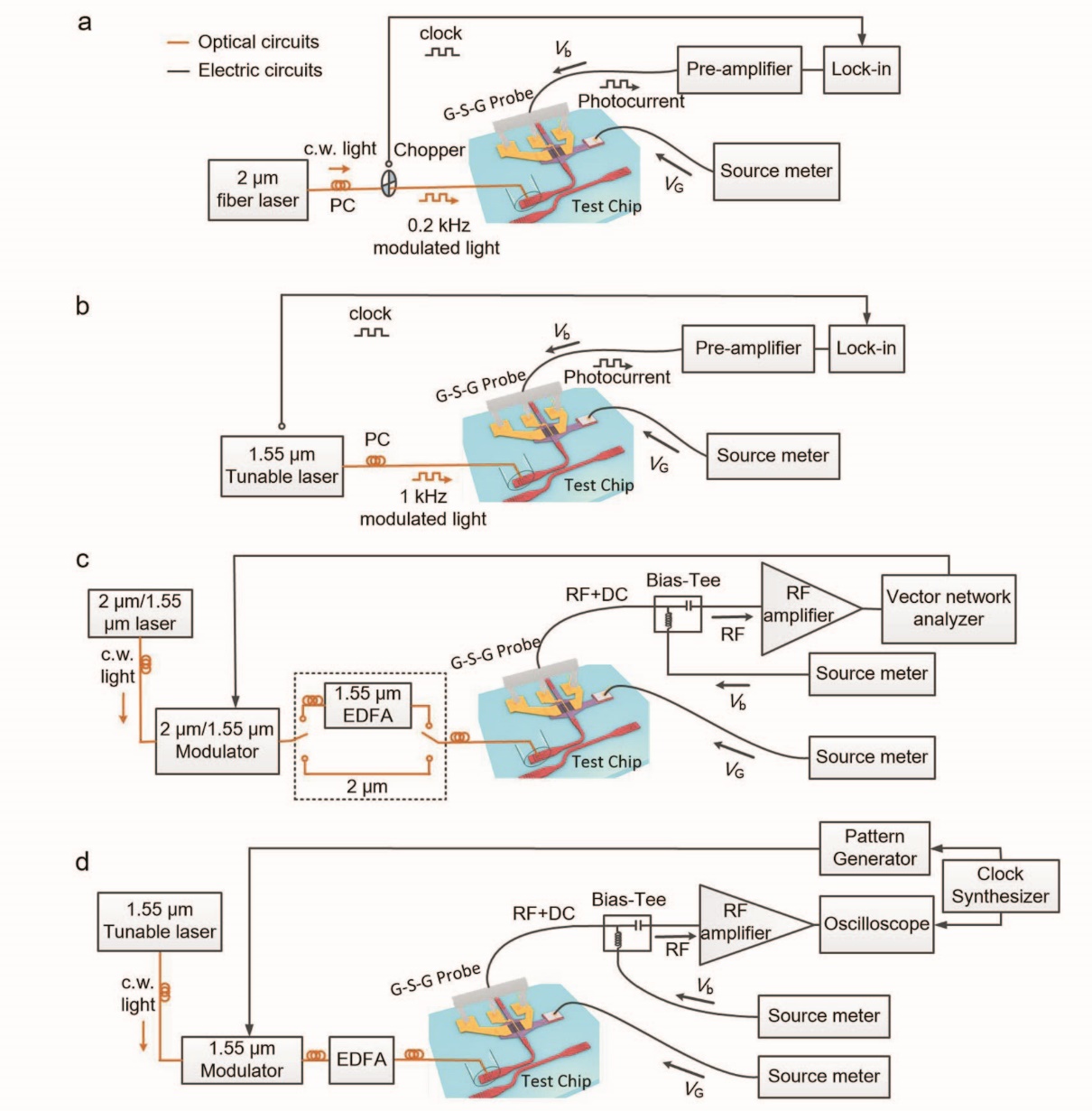


**Fig. S8** **Measurement setups.** **a** Low-frequency measurement setups for the devices when operating at 2 μm; **b** Low-frequency measurement setups for the devices when operating at 1.55 μm; **c** High-frequency measurement setups for the devices; **d** Eye-diagram measurement setups for the devices when operating at 1.55 μm. PC: Polarization controller.

Supplementary Figure S8c shows the experimental setup for measuring the high frequency response. The CW light was modulated with an optical modulator (2μm: IXBLUE MX2000-LN-10, 10 GHz bandwidth; 1.55 μm: Sumitomo T. MXH1.5DP-40PD-ADC, 22 GHz bandwidth), which was driven by the electrical signal from a vector network analyzer (ROHDE&SCHWARZ ZVA40, 40GHz). For the measurement of the 1.55 μm device, the modulated light was amplified by using an EDFA (Thorlabs EDFA100P) before it was coupled to the chip through an input grating coupler. For the measurement of the 2 μm device, no optical amplifier was available. The output electrical signal of the silicon-graphene waveguide photodetector was then amplified by using a RF amplifier (Centellax OA4MVM2) and finally received by the VNA. The calibrations were performed before the device tests with high-speed commercial photodetectors.

The eye diagram was measured with another test system, as shown in Fig. S8d. The PRBS signal generated by a pattern generator (Keysight N4952A) was used for driving the optical modulator. The electrical signal output from the silicon-graphene photodetector was amplified by a microwave system amplifier (Keysight N4985A) and finally received by a wide-bandwidth oscilloscope (Keysight DCA-X 86100D). The clock signal generated by Keysight N4960A was used for the synchronization of the pattern generator and the oscilloscope.

**Supplementary Note 8. Noise analysis.**

In Fig. S9, the on-off I-V curves of Devices A, B and C are given by extracting the photocurrent data from low-frequency measurements and the dark current data from the static measurement. It can be seen that the signal-to-dark-current ratios are low, as expected for MGM graphene photodetectors.

**

**

**Fig. S9** **The on-off I-V curves for the present graphene photodetectors. a** Device A, *P*_in_=2.2 mW; **b** Device B, *P*_in_=2.2 mW; **c** Device C, *P*_in_=0.63 mW.

Furthermore, we also give an analysis on the device sensitivity performance by evaluating the frequency-normalized noise equivalent power (NEP). The noise current mainly consists of the dark current shot noise current $\sqrt{\bar{i_{nd}^{2}}}$ and the Johnson current noise (thermal noise) current $\sqrt{\bar{i_{nJ}^{2}}}$ which satisfy

$\overline{i_{nd}^{2}}=2eI_{d}\Delta f\text{=}2e\Delta f\frac{\text{|}V_{b}|}{R_{tot}}$, (Eq. S12)

$\overline{i_{nJ}^{2}}=\frac{4k_{B}T\Delta f}{R_{tot}}$. (Eq. S13)

Here *I*_d_ is the dark current, *R*_tot_ is the total device resistance, ∆*f* is the bandwidth, *e* is the unit charge, *k*_B_ is the Boltzmann constant, and T = 300 K is the operating temperature. Then NEP is given by

$\text{NEP=}\frac{\left|i_{n}\right|/R_{es}}{\Delta f^{1/2}}\text{=}\frac{\sqrt{\overline{i_{nJ}^{2}}+\overline{i_{nd}^{2}}}/R_{es}}{\Delta f^{1/2}}=\frac{(4k_{B}T+2e\text{|}V_{b}\text{|})^{1/2}}{R_{es}R_{tot}^{1/2}}$, (Eq. S14)

where *R*_es_ is the device responsivity.

For Device A, the responsivity *R*_es_ is respectively 35~52 mA/W and 25.5~30 mA/W at *V*_bias_=0.3V when operating with the BOL and PC modes. The total resistance *R*_tot_ is respectively 93.4 Ω and 181.2 Ω. Then one has NEP=6.68~9.92×10^2^ pW/Hz^1/2^ and 61.7~72.7 pW/Hz^1/2^ for two operation modes, respectively.

For Device B, the responsivity *R*_es_ is 45~70 mA/W at *V*_bias_=0.3V, and the total resistance *R*_tot_ is 90.2 Ω. Correspondingly, one has NEP= 53.2~82.7 pW/Hz^1/2^.

For Device C operating at 1.55 μm, the responsivity *R*_es_ is 136~395 mA/W at *V*_bias_=0.3V, and the total resistance *R*_tot_ is 174.8 Ω. Correspondingly, one has NEP= 4.86~14.12 pW/Hz^1/2^.

# **Supplementary References**

1. Li, H. H. Refractive index of silicon and germanium and its wavelength and temperature derivatives, *J. Phys. Chem. Ref. Data* **9**, 561-658 (1993).
2. Malitson, I. H. Interspecimen comparison of the refractive index of fused silica, *J. Opt. Soc. Am.* **55**, 1205-1208 (1965).
3. Malitson, I. H. Refraction and dispersion of synthetic sapphire, *J. Opt. Soc. Am.* **52**, 1377-1379 (1962).
4. Rakić, A. D. et al. Optical properties of metallic films for vertical-cavity optoelectronic devices, *Appl. Opt.* **37**, 5271-5283 (1998).
5. Koester, S. J. & Li, M. Waveguide-coupled graphene optoelectronics. *IEEE J. Select. Topics Quantum Electron.* **20**, 84-94 (2014).
6. Chang, Z. & Chiang, K. S. Experimental verification of optical models of graphene with multimode slab waveguides. *Opt. Lett.* **41**, 2129-2132 (2016).
7. https://apps.lumerical.com/other_application_graphene_simulation_tips.html
8. Hanson, G. W. Dyadic Green’s functions and guided surface waves for a surface conductivity. *J. Appl. Phys.* **103**, 064302 (2008).
9. Huard, B. et al. Evidence of the role of contacts on the observed electron-hole asymmetry in graphene. *Phys. Rev. B* **78**, 121402 (2008).
10. Varykhalov, A.et al. Effect of noble-metal contacts on doping and band gap of graphene. *Phys. Rev. B* **82**, 121101 (2010).
11. Senanayake, P. et al. Surface plasmon-enhanced nanopillar photodetectors. *Nano Lett.* **11**, 5279-5283 (2011).
12. Xu, Y. et al. Contacts between two-and three-dimensional materials: ohmic, Schottky, and p–n heterojunctions. *ACS Nano* **10**, 4895-4919 (2016).
13. Urich, A., Unterrainer, K. & Mueller, T. Intrinsic response time of graphene photodetectors. *Nano Lett.* **11**, 2804-2808 (2011).
14. Walsh, E. D. et al. Graphene-based Josephson-junction single-photon detector. *Phys. Rev. Appl.* **8**, 024022 (2017).
15. Efetov, D. K. et al. Fast thermal relaxation in cavity-coupled graphene bolometers with a Johnson noise read-out. *Nat. Nanotechnol.* **13**,797 (2018).
16. Schall, D. et al. Graphene photodetectors with a bandwidth> 76 GHz fabricated in a 6 ″wafer process line. *J. Phys. D: Appl. Phys.* **50**, 124004 (2017).

Ma, P. et al. Plasmonically enhanced graphene photodetector featuring 100 Gbit/s data reception, high-responsivity and compact size. *ACS Photon.* **6**, 154-161 (2018).

1. Freitag, M. et al. Photoconductivity of biased graphene. *Nat. Photon.* **7**, 53 (2013).
2. Meric, I. et al. Current saturation in zero-bandgap, top-gated graphene field-effect transistors. *Nat. Nanotechnol.* **3**, 654 (2008).
3. Shiue, R. –J. et al. High-responsivity graphene–boron nitride photodetector and autocorrelator in a silicon photonic integrated circuit. *Nano Lett.* **15**, 7288 -7293 (2015).
4. Midrio, M. et al. Graphene based optical phase modulation of waveguide transverse electric modes. *Photon. Res.* **2**, A34–A40 (2014).
5. Yan, J. et al. Electric field effect tuning of electron-phonon coupling in graphene. *Phy. Rev. Lett.* **98**, 166802 (2007).
6. Mueller, T. et al. Role of contacts in graphene transistors: A scanning photocurrent study. *Phys. Rev. B* **79**, 245430 (2009).
7. Schuler, S. et al. Controlled generation of a p–n junction in a waveguide integrated graphene photodetector. *Nano Lett.* **16**, 7107-7112 (2016).
8. Low, T. et al. Cooling of photoexcited carriers in graphene by internal and substrate phonons. *Phys. Rev. B* **86**, 045413 (2012).


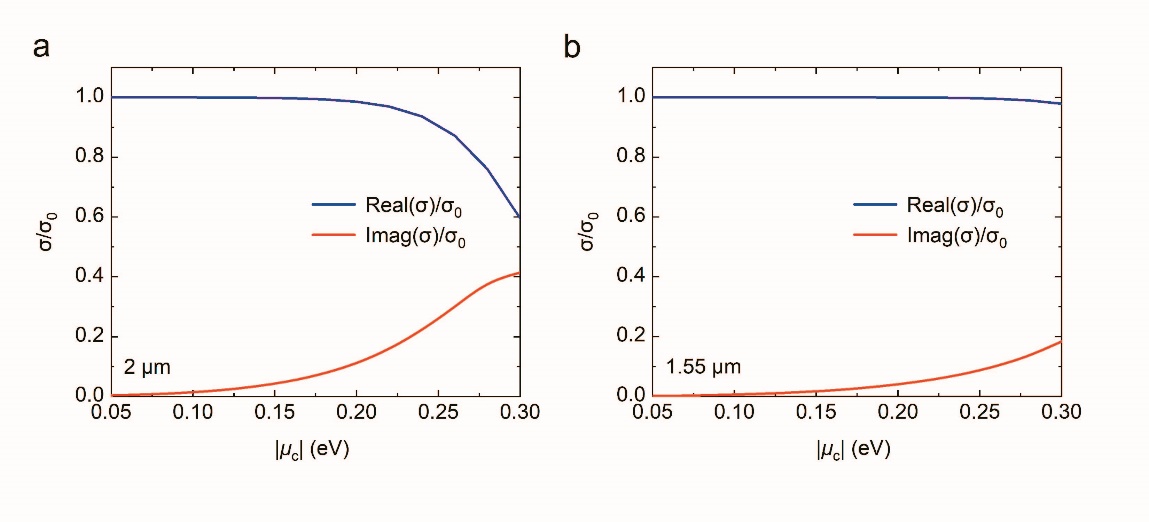


**Fig. S1** **The graphene optical conductivity versus the chemical potential. a** Wavelength is 2 μm; **b** Wavelength is 1.55 μm.


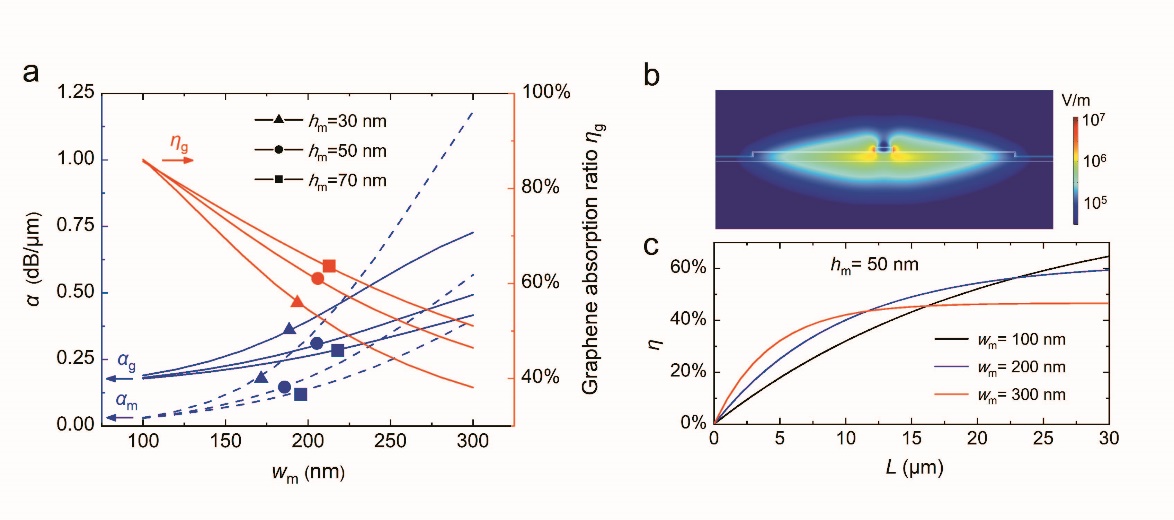


**Fig. S2** **Mode properties of the present silicon-graphene hybrid plasmonic waveguide when operating at λ=1.55 μm**. **a** Calculated absorption coefficients (*α*_g_, *α*_m­_)*,* and the graphene absorption ratio *η*_g_ as the metal-strip width *w*_m_ varies for the cases with different metal heights *h*_m_. Here *w*_si_=3 μm, and *h*_si_= 100 nm; **b** The electric field component $\sqrt{\left| \vec{E_{x}} \right|^{2}+\left| \vec{E_{z}} \right|^{2}}$ of the quasi-TE mode for the optimized silicon-graphene hybrid plasmonic waveguide (@ 1.55 μm); **c** Calculated graphene absorptance *η* as the propagation length *L* varies for the cases with different metal-strip widths *w*_m_ = 100, 200, and 300 nm. Here *h*_m_= 50 nm, *w*_si_= 3 μm, and *h*_si_= 100 nm.


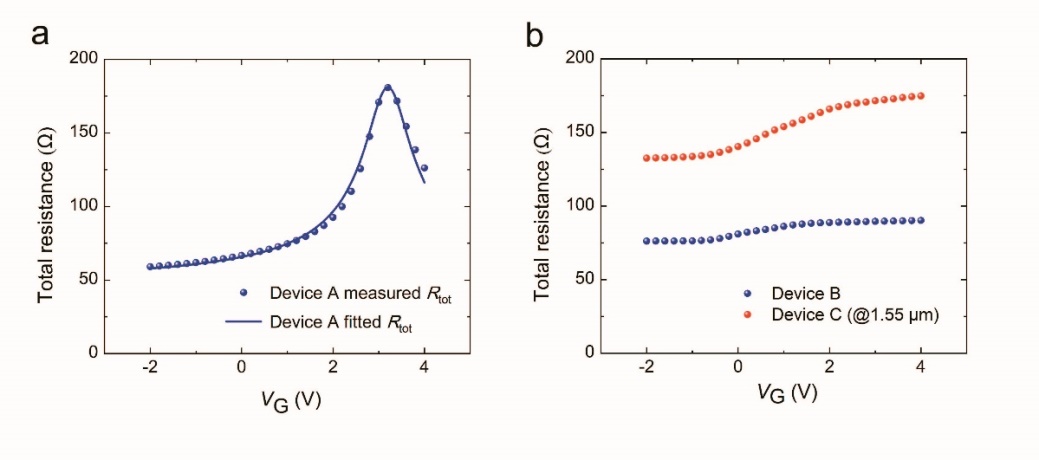


**Fig. S3** **Total device resistance *R*_tot_.** **a** Measured and fitted *R*_tot_ of Device A; **b** Measured *R*_tot_ for Devices B and C.


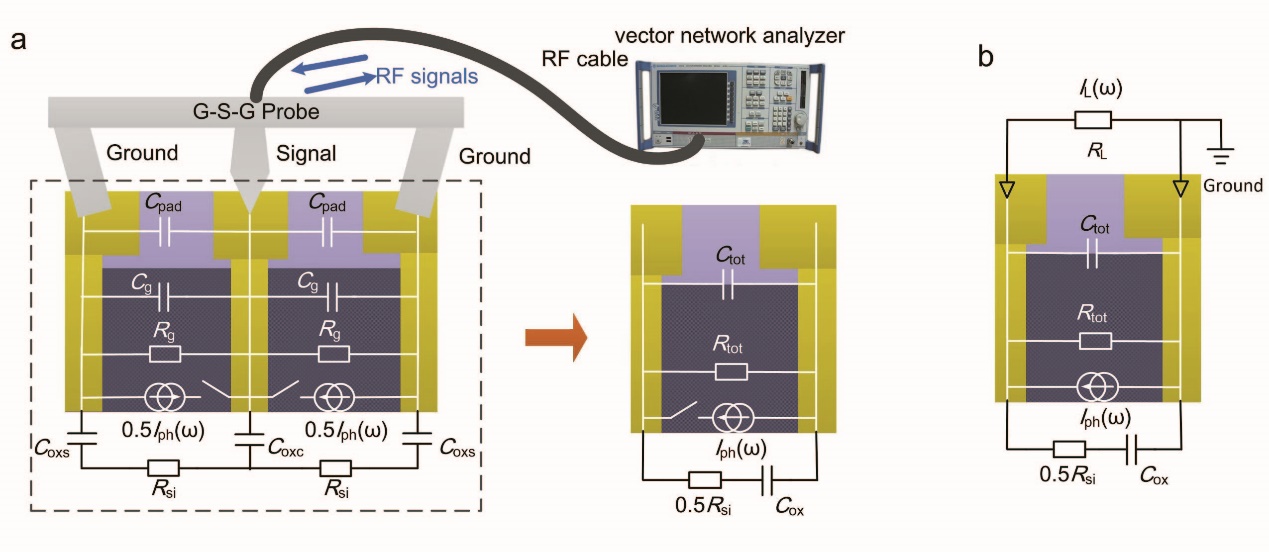


**Fig. S4** **Schematic diagrams of equivalent circuit model. a** Detailed and simplified equivalent circuits with the S_11_ measurement setup. **b** Simplified equivalent circuit for electric circuit frequency response S_21_^EC^ evaluation.


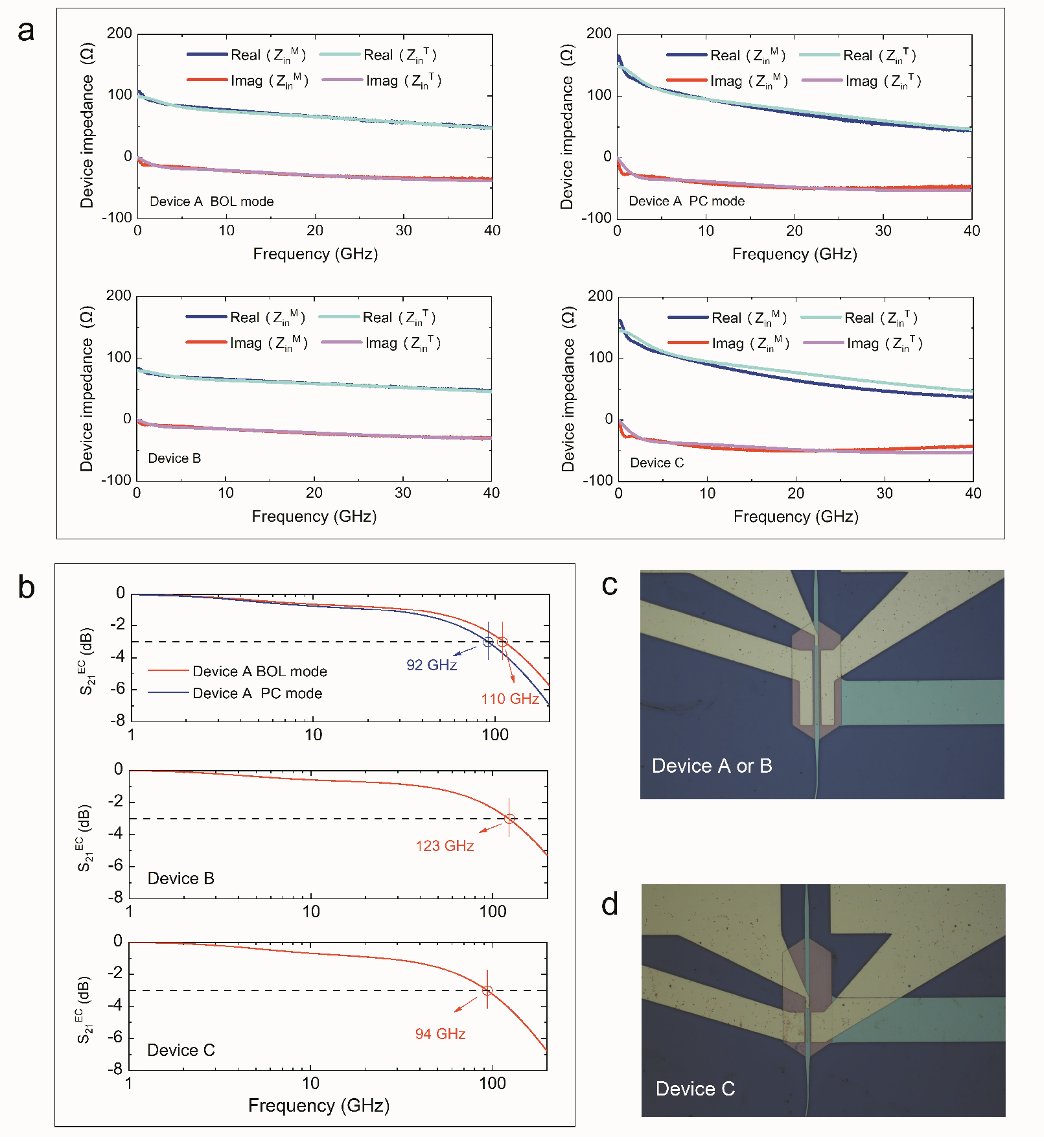


**Fig. S5** **Results of the equivalent circuit model. a** The measured impedances *Z*_in_^M^ and fitting impedances *Z*_in_^T^ given by real parts and imaginary parts for Device A at the BOL mode with *V*_G_=2.1V, Device A at the PC mode with *V*_G_=3.4V, Device B at the BOL mode with *V*_G_=2.9V, and Device C at the BOL mode with *V*_G_=2.8V; **b** The calculated electric circuit frequency response S_21_^EC^; **c** Structure of Device A or B; **d** Structure of Device C.


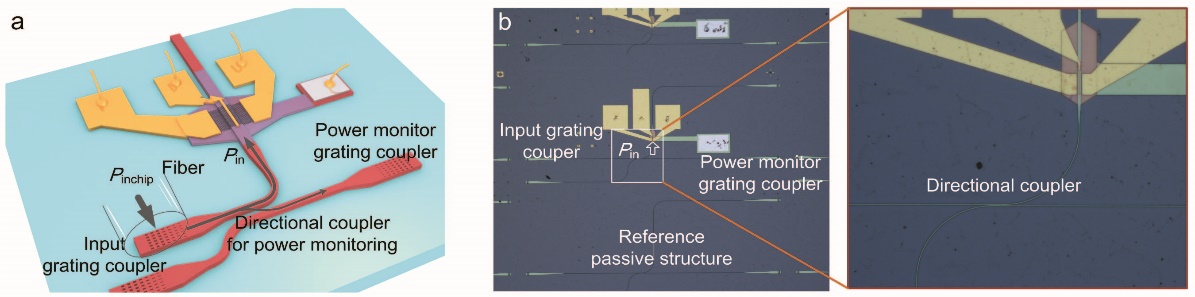


**Fig. S6** **Fabricated photonic integrated circuits.** **a** Schematic configuration; **b** microscopy pictures.


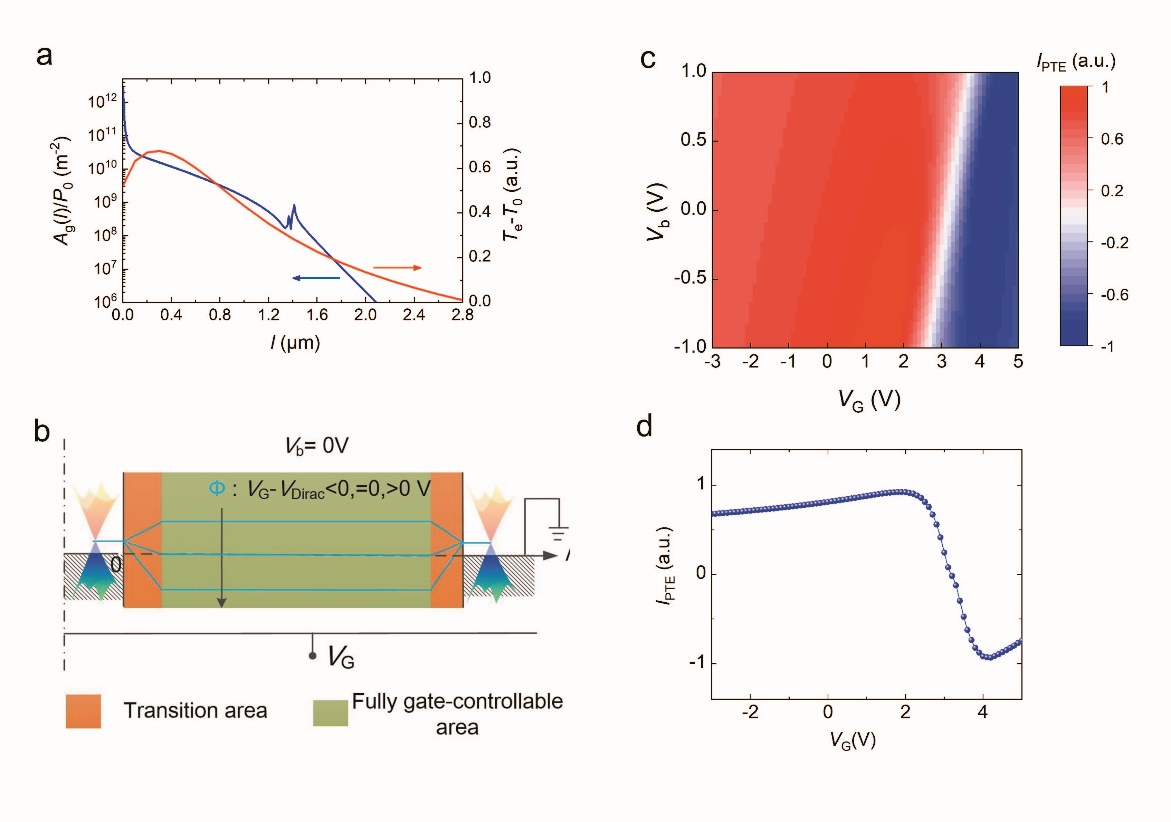


**Fig. S7** **The PTE photo-current modeling of Device A.** **a** Normalized graphene absorption density *P*(*l*) and average electron temperature increment ($\bar{T}_{e}-T_{0}$) with *V*_G_=*V*_Dirac_; **b** Energy-band diagram of the silicon-graphene photodetector with *V*_b_=0; **c** PTE photo-current as the voltages *V*_b_ and *V*_G_ varies; **d** PTE photo-current versus *V*_G_ when *V*_b_=0.


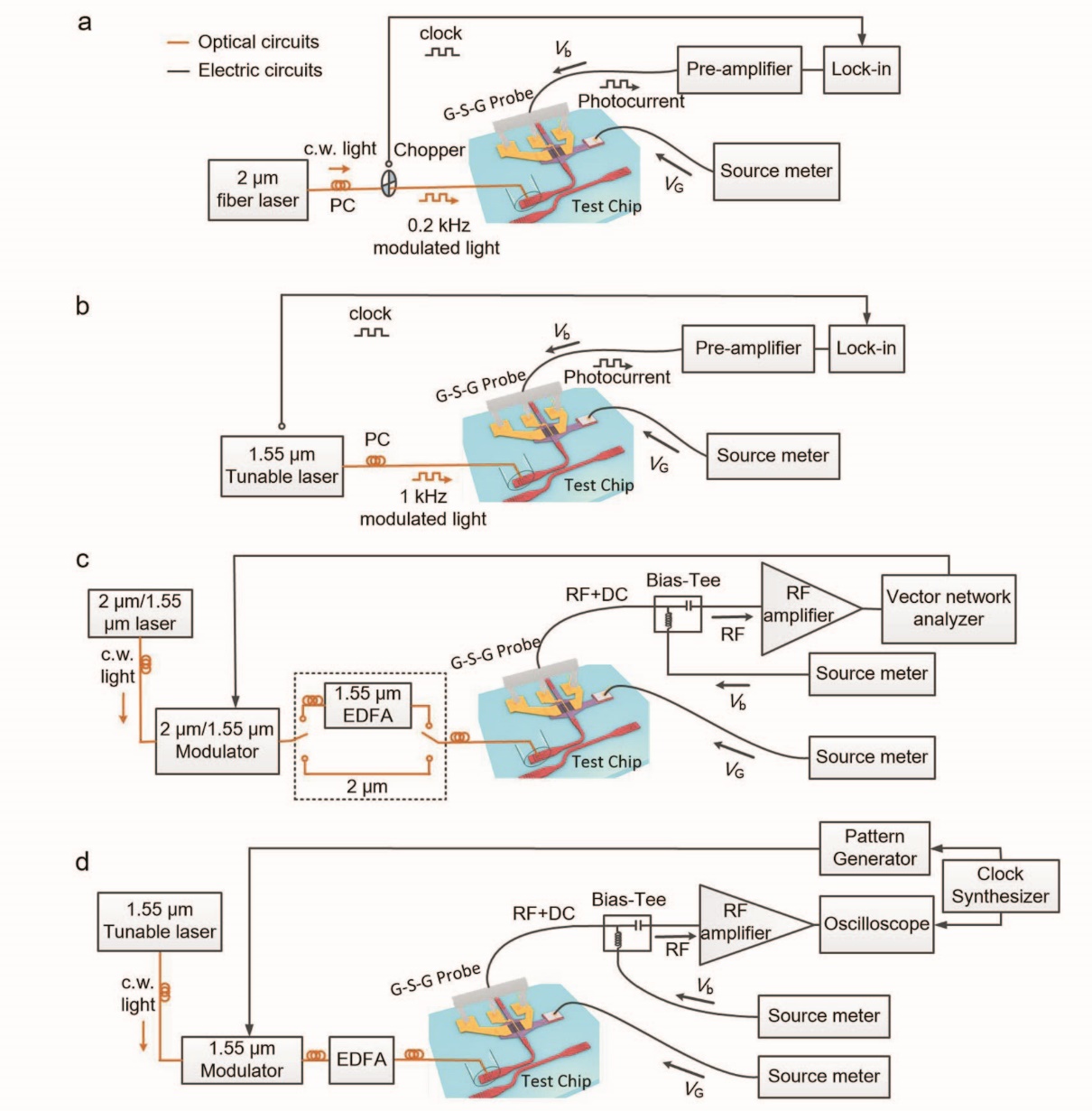


**Fig. S8** **Measurement setups.** **a** Low-frequency measurement setups for the devices when operating at 2 μm; **b** Low-frequency measurement setups for the devices when operating at 1.55 μm; **c** High-frequency measurement setups for the devices; **d** Eye-diagram measurement setups for the devices when operating at 1.55 μm. PC: Polarization controller.

**

**

**Fig. S9** **The on-off I-V curves for the present graphene photodetectors. a** Device A, *P*_in_=2.2 mW; **b** Device B, *P*_in_=2.2 mW; **c** Device C, *P*_in_=0.63 mW.

**Table S1. The parameters for the equivalent circuit extracted from the measured S_11_ and the estimated electric-circuit-limited 3dB-bandwdith BW_RC-3dB_.**

| Devices | Mechanism | *R*_tot_  (Ω) | 0.5·*R*_si_  (Ω) | *C*_tot_  (fF) | *C*_ox_  (fF) | BW_RC-3dB_  (GHz) | *L*_g_  (μm) | *W*_g_  (μm) | *λ*  (μm) |
| --- | --- | --- | --- | --- | --- | --- | --- | --- | --- |
| A | BOL effect | 98.6 | 370.8 | 39.7 | 95.2 | 110 | 50 | 2.8 | 2 |
|  | PC effect | 148.5 | 362.2 | 41.4 | 97.8 | 92 |  |  |  |
| B | BOL effect | 79.8 | 375.8 | 38.8 | 97.1 | 123 |  |  |  |
| C | BOL effect | 145.6 | 370.1 | 40.5 | 79.2 | 94 | 20 | 2.2 | 1.55 |
